# The global proteome of *Streptococcus pneumoniae* EF3030 under nutrient-defined *in vitro* conditions

**DOI:** 10.3389/fcimb.2025.1606161

**Published:** 2025-07-11

**Authors:** Supradipta De, Larissa M. Busch, Gerhard Burchhardt, Manuela Gesell Salazar, Rabea Schlüter, Leif Steil, Uwe Völker, Sven Hammerschmidt

**Affiliations:** ^1^ Department of Molecular Genetics and Infection Biology, Interfaculty Institute of Genetics and Functional Genomics, University of Greifswald, Greifswald, Germany; ^2^ Department of Functional Genomics, Interfaculty Institute of Genetics and Functional Genomics, University Medicine, Greifswald, Germany; ^3^ Imaging Center of the Department of Biology, University of Greifswald, Greifswald, Germany

**Keywords:** *Streptococcus pneumoniae*, minimal growth medium, infection related condition, bacterial morphology, scanning electron microscopy, proteome analysis, competence

## Abstract

**Introduction:**

*Streptococcus pneumoniae* is a human pathobiont that asymptomatically colonizes the upper respiratory tract but can cause severe diseases such as pneumonia, sepsis, and meningitis, as well as non-invasive infections like otitis media and sinusitis. It thrives in the nutrient-limited environment of the nasopharynx and has evolved mechanisms to manage host-induced stress and regulate protein levels accordingly.

**Methods:**

To investigate the molecular biology of *S. pneumoniae* under in vitro and infection-relevant conditions, a suitable cultivation medium is essential for reproducible experiments. We, therefore optimized a chemically defined minimal medium that mimics the nutrient-limited conditions of the human nasopharynx. This medium was used to cultivate clinical isolates and other streptococcal species for proteomic analysis.

**Result:**

The optimized medium enhanced growth and shortened the lag phase of *S. pneumoniae* and related species. Using this medium, we analyzed the global proteome of the pneumococcal colonizing strain EF3030 during its transition from early to late logarithmic growth phase. Distinct changes in protein abundance were observed in functional categories such as metabolism, amino acid synthesis, natural competence, RNA and cell wall synthesis, protein degradation, and stress responses. Notably, proteins involved in DNA uptake and processing—such as choline-binding protein CbpD, competence factors ComGA and ComEA, and ssDNA-binding proteins Dpr and DprA—were more abundant in the late log phase.

**Discussion:**

These findings highlight dynamic proteomic changes associated with pneumococcal adaptation to nutrient-limited conditions and provide insights into the biology of strain EF3030 during colonization. The optimized medium offers a reproducible platform for studying pneumococcal physiology and pathogenesis under defined conditions.

## 1 Introduction

*Streptococcus pneumoniae*, also known as pneumococcus, exhibits a range of growth behaviors, including planktonic growth as diplococci, in chains, or in biofilms. Biofilm formation is particularly significant in the asymptomatic colonization of the upper respiratory tract (URT), where *S. pneumoniae* predominantly resides (Chao et al., 2014; Gilley and Orihuela, 2014). However, any alteration in the URT can trigger the dissemination of pneumococci and the onset of pathogenesis (Simell et al., 2012). The ability of pneumococci to cross host tissue barriers is a critical factor in its potential to cause both non-invasive and invasive diseases such as otitis media, sinusitis, pneumonia, septicaemia, or meningitis, posing significant global health challenges (Bogaert et al., 2004; Yao and Yang, 2008; Hammerschmidt, 2006; Cartwright, 2002).

The growth of *S. pneumoniae* is significantly influenced by a diverse array of environmental and nutrient factors that can vary widely between host compartments and controlled growth conditions (Härtel et al., 2012; Schulz et al., 2014; Carvalho et al., 2013; Leonard et al., 2018; Sanchez-Rosario and Johnson, 2021; Burghout et al., 2013; Fatykhova et al., 2024; Schulz and Hammerschmidt, 2013; Härtel et al., 2011). Carbohydrates and amino acids, particularly, play a decisive role in the success of the bacteria in encountering and adapting to different host niches, and in causing infectious diseases (Härtel et al., 2012). Within its primary niche, the URT, the availability of nutrients, including nasal transition metal levels, differs substantially from other environments such as bloodstream and lungs, which influences the phenotype and pathophysiology of pneumococci (van Beek et al., 2020; Krismer et al., 2014; De and Hakansson, 2023; Im et al., 2022).

A previous study by our group (Schulz et al., 2014) introduced several supplements to RPMI 1640, including uracil, adenine, glycine, choline chloride, phosphate, carbonate, and glucose, to improve the cultivation of streptococcal strains in a minimal medium. Notably, nasal fluid contains 10-fold higher concentrations of calcium and magnesium compared to iron, copper and zinc (van Beek et al., 2020). These variations in metal availability have a profound impact on *S. pneumoniae* growth and metabolism, for example, the balance between iron and manganese plays a key role in regulating peroxide levels via the Fenton reaction, thereby influencing the oxidative stress response and contributing to pneumococcal pathogenicity (Ong et al., 2013). Understanding host compartment-specific nutrient variations provides valuable insights into bacterial metabolism and the mechanisms that enable *S. pneumoniae* to adapt to different environments, thereby improving *in vitro* models that more accurately mimic the host environment.

In the nasopharynx, *S. pneumoniae* encounters significant challenges in overcoming mucus entrapment and evading the immune defense systems of the host. The capsular polysaccharide (CPS) helps pneumococci to bypass the electrostatic repulsion from mucus, facilitating bacterial adhesion to epithelial cells (Nelson et al., 2007). However, the capsule can also conceal key surface proteins involved in recognizing host cells. To overcome this, *S. pneumoniae* can undergo phase variation, switching to transparent variants that express lower capsule amounts and higher surface proteins (Weiser and Kapoor, 1999). Moreover, when in close contact with host cells, pneumococci reduce capsule levels, exposing adhesins that enhance interaction (Hammerschmidt, 2006). *S. pneumoniae* also employs exoglycosidases such as neuraminidase (NanA, NanB, and NanC), β-galactosidase (BgaA), and StrH, to modify host carbohydrate structures, thereby promoting adhesion (King, 2010). Recently, the concerted action of neuraminidases and pneumolysin in the destruction of the platelets was shown. Pneumococcal neuraminidases desialylate platelet glycoproteins, thereby increasing binding of pneumolysin, which finally leads to high cytotoxicity even at low toxin concentrations (Fritsch et al., 2024). Furthermore, pneumococci exploit host proteases such as plasmin, to degrade mucosal and extracellular matrix (ECM) components, facilitating stronger interactions with host cells (Bergmann and Hammerschmidt, 2007). Key surface adhesins including PspC, PavA, PavB, enolase, and pili allow *S. pneumoniae* to bind to host receptors and ECM proteins, promoting attachment and colonization (Weiser et al., 2018; Jensch et al., 2010; Elm et al., 2004). PavA and PavB target and bind to different regions of fibronectin to enhance adhesion to host cells (Kanwal et al., 2017). Lipoproteins such as PnrA, DacB, and MetQ, along with surface proteins like PspA, PspC, and PsaA, are promising candidates for vaccine development due to their role in adhesion and immune evasion (Narciso et al., 2024; Paulikat et al., 2024; Voß et al., 2018; Kohler et al., 2016). Additionally, the Pht proteins, especially PhtD and PhtE, are crucial for adherence and colonization, making them valuable targets for therapeutic intervention (Khan and Pichichero, 2012; Kallio et al., 2014).In addition to the proteins mentioned earlier, proteases and peptidases also play a crucial role in bacterial adaptation to host environments and immune evasion (Marquart, 2021). These enzymes enable *S. pneumoniae* to degrade host tissues, modulate immune responses, and survive in nutrient-limited conditions commonly encountered during infection (Ali et al., 2021b; Bergmann and Hammerschmidt, 2006). Proteomic analyses have identified key enzymes whose abundance fluctuates during bacterial growth, potentially influencing the bacterium’s ability to transition between colonization and invasive phases (Lee et al., 2006). The regulation of enzyme abundances under different growth conditions, particularly during stress, highlights the significance of these enzymes in the pathophysiology of *S. pneumoniae* (Lee et al., 2006).

Due to the limitations of infection-related and nutrient-defined *in vitro* media suitable to study the pathophysiology of pneumococci, we developed an optimized and user-friendly minimal medium in the present study. We then utilized this medium to elucidate the global proteome landscape of the non-invasive strain *S. pneumoniae* EF3030.

## 2 Methodology

### 2.1 Bacterial strains and growth conditions

The bacterial strains used in this study (**Supplementary Table S1**) were initially retrieved from glycerol stocks and cultured on blood agar plates (BA), consisting of Columbia agar supplemented with 5% sheep blood. Following this, the strains were re-streaked onto fresh BA plates and incubated for a maximum of 8-10 hours at 37°C in the presence of 5% CO_2_. Bacteria from these BA plates were then used to inoculate the liquid medium.

To compare growth, bacterial strains were grown in RPMI-based chemically-defined medium (CDM) as established by (Schulz et al., 2014) to ensure reproducible growth, addressing its limitations in supporting proper growth for certain *S. pneumoniae* serotypes. The medium was further modified by supplementing it with methionine, iron and manganese, resulting in CDM+ (**Tables 1**–**3**).

**Table 1:** Stock solutions for modified CDM medium.

Solutions	Components	Concentration [g/l]	Remarks
Buffer solution	NaHCO_3_	24.7	sterile filter and store at 4˚C
	Glycine	1.11	
	Choline chloride	0.456	
	NaH_2_PO_4_ · H_2_O	3.195	
	Na_2_HPO_4_ · 2H_2_O	9.22	
	Glucose	74	
Adenine/Uracil solution	Adenine	4	dissolve Adenine and Uracil in 40ml 1M HCl at 90˚C, then adjust the total volume with distilled water, sterile filter and store at room temp.
	Uracil	8	
Iron and Manganese solution	MnSO_4_ · H_2_O	0.7	
	FeSO_4_ · 7H_2_0	0.625	dissolve in sterile distilled water and store at 4˚C
	Fe(NO_3_)_3_ · 9H_2_O	0.125	

**Table 2:** Preparation of modified RPMI 1640-based CDM**+** medium.

Components	Volume from the stock [ml]	Remarks
Buffer solution	40.5	
Adenine/Uracil solution	5	re-heat the solution, stirring at 90˚C
Iron and manganese solution	4	
Methionine solution (1mg/ml)	1	sterile filter and store at 4˚C

The supplements are directly added to 500ml RPMI-1640 medium with glutamine (HyClone™, Germany). The solutions are mixed and stored at 4˚C.

**Table 3:** Difference between the CDM and CDM+ medium.

Solutions	Components	Concentration [G/L]	CDM	CDM+
Buffer solution	NaHCO_3_	24.7	✓	✓
	Glycine	1.11	✓	✓
	Choline chloride	0.456	✓	✓
	NaH_2_PO_4_ · H_2_O	3.195	✓	✓
	Na_2_HPO_4_ · 2H_2_O	9.22	✓	✓
	Glucose	74	✓	✓
Adenine/Uracil solution	Adenine	4	✓	✓
	Uracil	8	✓	✓
Iron and Manganese solution	MnSO_4_ · H_2_O	0.7	-	✓
	FeSO_4_ · 7H_2_0	0.625	-	✓
	Fe(NO_3_)_3_ · 9H_2_O	0.125	-	✓
Methionine solution		0.0018	-	✓

For *S. pneumoniae, S. suis, S. mutans* and *S. agalactiae* pre-cultures were inoculated with an initial optical density (OD_600nm_) of 0.2 in CDM or CDM+. The strains were then sub-cultivated in fresh, pre-warmed minimal medium when they reached an optical density of 0.4, ensuring highly reproducible growth conditions during experiments. *S. pyogenes* was cultivated without subculturing, as the culture did not reach an optical density (OD) of 0.4. The growth of the other bacterial strains was monitored from an initial OD_600nm_ 0.2. For electron microscopic analysis, cultivation of pneumococcal strains was done in Todd-Hewitt broth with 0.5% yeast extract (THY). The strains were inoculated at an optical density (OD_600nm_) of 0.08.

### 2.2 Field emission scanning electron microscopy (SEM) of pneumococcal strain

Pneumococcal strains were cultured in THY, CDM or CDM**+** as described above, and samples were collected at an OD_600nm_ of 0.4 to 0.6. Bacteria were harvested by centrifugation at 3500 rpm, washed with phosphate-buffered saline (PBS), and fixed with 2.5% glutaraldehyde and 2.0% paraformaldehyde in washing buffer (0.1 M cacodylate buffer [pH 7], 0.09 M sucrose, 0.01 M CaCl_2_, 0.01 M MgCl_2_) for 1 hour at 4°C. Following fixation, pneumococci were immobilized to poly-L-lysine-coated coverslips for 90 minutes at room temperature, and fixation continued at 4°C overnight. The samples were then washed three times with washing buffer for 5 minutes each time, followed by treatment with 1% osmium tetroxide in washing buffer for 1 hour at room temperature. After three additional washing steps with washing buffer for 5 minutes each time, the samples were dehydrated in a graded series of aqueous ethanol solutions (10%, 30%, 50%, 70%, 90%, 100%) on ice for 15 minutes at each concentration. Prior to the final change to 100% ethanol, the samples were allowed to reach room temperature and were then critical point-dried using liquid CO_2_. Finally, the samples were mounted onto aluminium stubs, sputter-coated with gold/palladium, and examined with a field emission scanning electron microscope Supra 40VP (Carl Zeiss Microscopy Deutschland GmbH, Oberkochen, Germany) using the Everhart-Thornley SE detector and the inlens detector at a 80:20 ratio at an acceleration voltage of 5 kV. All micrographs were edited using Adobe Photoshop CS6 (**Supplementary Figure S1**).

### 2.3 Preparation of *S. pneumoniae* cellular and supernatant proteins for proteome studies

#### 2.3.1 Sample harvesting

*S. pneumoniae* strain EF3030 (serotype 19F) was cultured in CDM**+** with sub-cultivation as described above. Pneumococci were harvested at three defined optical densities: early exponential phase (OD_600_ 0.4), mid exponential phase (OD_600_ 0.6), and late exponential phase (OD_600_ 1.0). A total of 6 mL of culture was centrifuged at 3900 rpm for 10 minutes, and the supernatant was carefully collected and the tube was immediately frozen by immersion in ice-cold ethanol and maintained at -70°C. The bacterial pellet was washed once with PBS, and the optical density was adjusted to 1.0. Subsequently, 1 mL of the bacterial suspension was centrifuged again at 3900 rpm for 5 minutes, and the resulting bacterial pellet was stored at -70°C until sample preparation.

#### 2.3.2 Sample preparation

The collected samples of bacterial pellets were each resuspended in 50 µL of 20 mM HEPES buffer (pH 8) containing 1% SDS. Cell disruption was achieved through mechanical disruption using a bead mill at 3000 rpm for 3 minutes in liquid nitrogen. The resulting frozen powder was then dissolved in 150 µL of 20 mM HEPES buffer (pH 8) with 1% SDS. The solution was subjected to shaking at 1400 rpm at 95°C for 1 minute. After cooling, Pierce™ Universal Nuclease (Pierce, Thermo Fisher Scientific, MA, United States; 2.5 U, 4 mM MgCl2) was added, followed by an incubation in an ultrasonic bath for 5 minutes. Thereafter, all samples were centrifuged at 17000 g for 30 minutes, and the supernatant was carefully collected for subsequent analysis by mass spectrometry.

Proteins from the supernatant were precipitated using trichloroacetic acid (TCA). A volume of 1.5 mL of the supernatant was mixed with 225 µL of 100% TCA, resulting in a final concentration of 15% TCA. The solution was thoroughly mixed and incubated at 4°C for 48 hours. Following incubation, the solution was centrifuged at 17,000 g for 60 minutes at 4°C. The supernatant was carefully discarded, and the pellet was washed with 70% ice-cold ethanol by centrifugation at 17,000 g for 10 minutes at 4°C. This wash procedure was repeated three times with decreasing volumes of ethanol: 500 µL wash 1), 200 µL (wash 2), 180 µL (wash 3). After the final washing step, the ethanol was removed and the pellet containing the proteins was air-dried. The proteins were then resuspended in 15 µL of 20 mM HEPES buffer (pH 8) containing 1% SDS to solubilize the membrane proteins and incubated at 65°C for 2 minutes. Protein concentrations of protein samples were determined using the Thermo Micro BCA Kit employing the BCA assay analysis pipeline included in the MassSpecPreppy pipeline (Reder et al., 2024). Tryptic digestion of the pellet and supernatant-derived proteins was performed using the SP3 protocol (Reder et al., 2024). The resulting peptides were analyzed by mass spectrometry on an Orbitrap Exploris™ 480 mass spectrometer (Thermo Fisher Scientific), coupled to an Ultimate 3000 nano-LC system (Thermo Fisher Scientific). A data-independent acquisition (DIA) mode was utilized to acquire the data, for specific details see **Supplementary Tables S3** and **S4**. Data are available at ProteomeXchange consortium (Deutsch et al., 2023) via PRIDE repository with identifier PXD062379 (Perez-Riverol et al., 2024, 2016).

#### 2.3.3 Data analysis

The mass spectrometric data were analyzed using the SpectronautTM Software (v19.1), via a spectral library-based approach. The search against a protein sequences database of *S. pneumoniae* EF3030 (NCBI RefSeq accession no. NZ_CP035897.1, alternatively: NZ_CP035897) included tryptic peptides with up to two missed cleavages, fixed carbamidomethylation of cysteine and oxidation of methionine and N-terminal acetylation as variable modifications. Further analysis was performed using R version 4.4.1 and the SpectroPipeR package 0.3.0 (Michalik et al., 2025). The PneumoWiki (https://pneumowiki.med.uni-greifswald.de) annotations and descriptions of *S. pneumoniae* EF3030 proteins were used to analyze the genome’s functional response to the minimal medium.

For comparative analysis of protein composition between the cellular and supernatant fractions, intensity-based absolute quantification (iBAQ) values were calculated using Spectronaut. The relative iBAQ values were expressed as a percentage of the total sum of iBAQ values for each sample condition (Schwanhäusser et al., 2011) (**Supplementary Figure S3**). Protein localization were predicted using DeepLocPro v1.0 (Moreno et al., 2024) with ‘−−group positive’ Gene set enrichment analyses (GSEA; Subramanian et al., 2005) was performed using the fast gene set enrichment analysis (fgsea) R-package (Korotkevich et al., 2016), leveraging TIGRFAM (Haft et al., 2013) functional annotations of *S. pneumoniae* EF3030 proteins as retrieved from PneumoWiki and integrating orthologously mapped RegPrecise (Novichkov et al., 2013) based regulon information of *S. pneumoniae* TIGR4 (**Supplementary Figure S2**). Hierarchical clustering of protein profiles of proteins identified with at least two peptides in both fractions was performed in R using the hclust function (**Supplementary Excel Table S3**). As distance metric, spearman correlation was applied, and 1-r was used. For distance determination between clusters, the average method was used. Clusters cutting height was set to 40% of the maximal distance. Functional characterization of the clusters was performed by over representation analysis using the Fisher’s Exact test and employing TIGRFAM functional annotation and regulon information.

## 3 Results

### 3.1 Methionine and the trace elements, iron and manganese enhanced streptococcal fitness and growth in minimal medium

**Figure 1:**
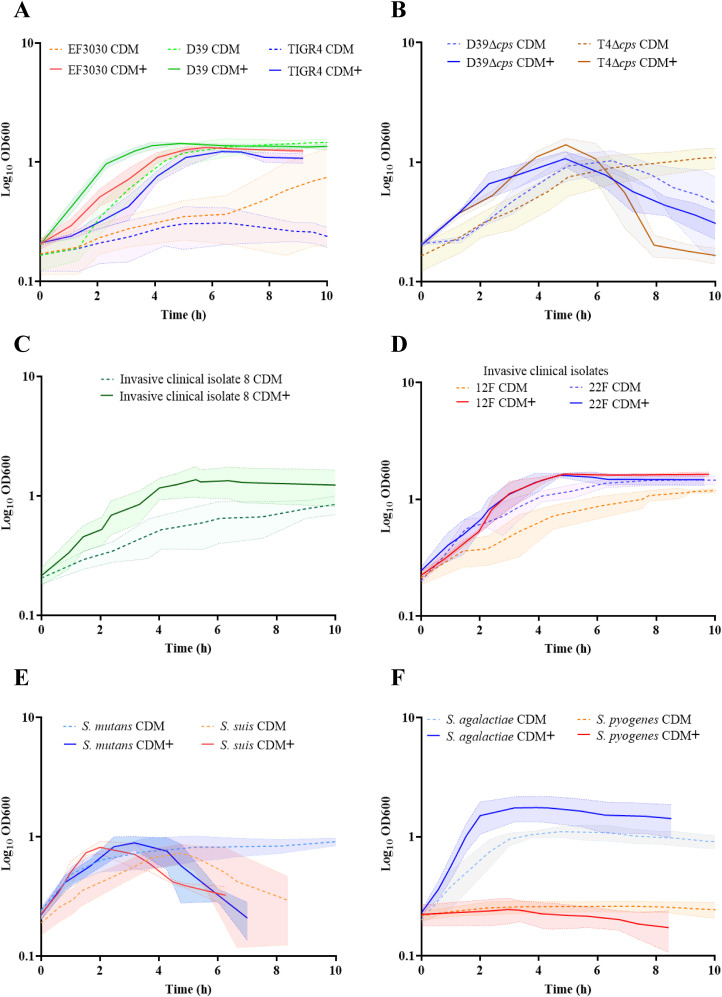
Optimization of a minimal medium for growth of pneumococcal and other streptococcal species. To establish a reliable and reproducible growth medium for pneumococcal and other streptococcal species, we compared the growth of these bacteria in two different media. Bacteria were cultured in RPMI supplemented with adenine and uracil (CDM), represented by the dotted lines, and in RPMI supplemented with adenine, uracil, methionine, iron, and manganese (CDM+), represented by the bold lines. The standard deviation (SD) of the measurements is represented by the shadow, indicating the variability of the results across three independent replicates (n=3). **(A)** Growth of pneumococcal strains, serotype 19F (EF3030), serotype 2 (D39) and serotype 4 (TIGR4). **(B)** Nonencapsulated mutants: D39Δ*cps* and TIGR4Δ*cps.* **(C)** Invasive clinical isolates of *S. pneumoniae*: serotype 8. **(D)** Invasive clinical isolates of *S. pneumoniae*: 12F and 22F. **(E)** *S. mutants* and *S. suis.* **(F)** Group A streptococcal strain, *S. pyogenes* and Group B strain, *S. agalactiae*.

Previous studies have shown that amino acids are essential for improved growth behavior in minimal medium for the cultivation of *S. pneumoniae*, serotype 2, D39 (Härtel et al., 2012). Later a RPMI based chemical-defined minimal medium was established with supplementation of uracil-adenine solution (CDM) (Schulz et al., 2014). However, *S. pneumoniae* EF3030, serotype 19F, and TIGR4, serotype 4, exhibited a prolonged lag phase and a lower yield in this medium (Ali et al., 2021a). To further improve the growth conditions and ensure reproducibility of pneumococcal growth, we supplemented CDM with methionine as well as with the metal ions, iron and manganese, which are needed as trace elements for bacterial growth. This newly developed minimal medium (CDM**+**) significantly reduced the lag phase of the strain EF3030. Similarly, growth of the other laboratory strains TIGR4, D39 and capsule knockout strains, D39Δ*cps* and TIGR4Δ*cps* was enhanced in CDM**+** (**Figures 1A, B**). Furthermore, we cultivated pneumococcal clinical isolates of serotype 8, 12F, or 22F in CDM**+** and monitored their growth. Indeed, a shorter lag phase and accelerated growth were observed for all strains (**Figures 1C, D**). To assess growth of other pneumococcal strains in CDM+, we tested a larger panel of invasive clinical isolates covering some of the serotypes included in the PPSV-23 vaccine (**Supplementary Figure S4** and **Supplementary Table S1**). To test whether this medium is also suitable for cultivating other streptococcal species, we tested a panel of streptococcus species that are not only phylogenetically and physiologically diverse, but also clinically and scientifically significant. We cultivated *S. mutans, S. suis*, *S. pyogenes* (GAS) and *S. agalactiae* (GBS) in CDM**+** (**Figures 1E, F**). However, *S. mutans* and *S. suis* strains showed similar growth in both media, CDM and CDM**+** (**Figure 1E**). Remarkably, *S. pyogenes* showed no real growth in both media but also no death phase, suggesting that this fastidious organism has more stringent growth requirements and may require additional components for growth in a minimal medium. In contrast, *S. agalactiae* grew to higher optical density in CDM**+** (**Figure 1F**). Since this medium supports growth of clinically and biologically important streptococci and pneumococci, we conclude that the improved minimal medium CDM**+** can be applied to study the (patho-)physiology of different streptococcal species under reliable growth conditions.

### 3.2 Morphological characteristics of pneumococci in the exponential growth phase

**Figure 2:**
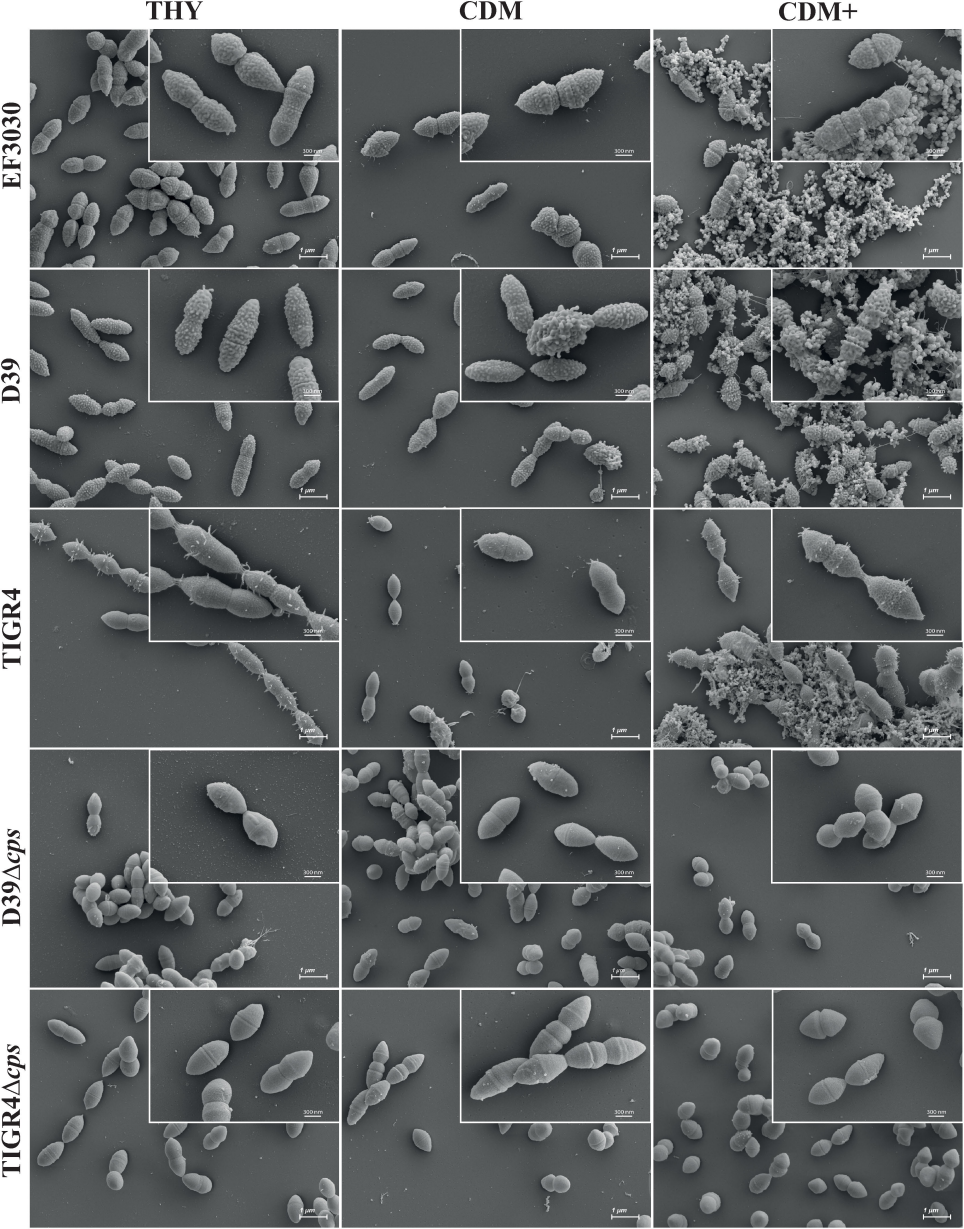
Scanning electron micrographs of *S. pneumoniae* cultivated in THY, CDM, or CDM**+**. EF3030 (serotype 19F), D39 (serotype 2), TIGR4 (serotype 4), D39Δ*cps* and TIGR4Δ*cps* were cultured at 37˚C up to an optical density of OD_600nm_ 0.6 in each medium. Pneumococci were harvested and subjected to scanning electron microscopy. The micrographs were taken at a magnification of 10,000x and the inset micrographs in the top right corner were taken at a higher magnification of 30,000x. These higher magnifications provide more detail of the pneumococcal surface structures and aggregates. Scale bars = 1 µm; Scales bars of the inset micrographs = 300 nm (see **Supplementary Figure S1**).

Three encapsulated pneumococcal strains (D39, TIGR4, and EF3030) of different serotypes (2, 4, and 19F respectively) along with the isogenic non-encapsulated mutants (TIGR4Δ*cps* and D39Δ*cps*) were cultivated in three different media: the complex medium THY, as well as the minimal media CDM and CDM**+**. Pneumococci were harvested in exponential growth phase at an optical density of (OD_600nm_) 0.6 and prepared for scanning electron microscopy to visualize and illustrate the organization and morphology of the pneumococcal strains at high resolution. The encapsulated strains EF3030, TIGR4, and D39 formed aggregates in CDM**+**, whereas they did not form these assemblages when cultivated in either CDM or THY (**Figure 2**, **Supplementary Figure S1**), suggesting that the bacterial aggregates are due to the supplements present in CDM**+**. In contrast to wild-type pneumococci, the isogenic non-encapsulated strains TIGR4Δ*cps* and D39Δ*cps* appear smooth due to the lack of capsule and did not form substantial aggregates even in CDM**+**, suggesting an impact of the capsular polysaccharide (CPS) on pneumococcal aggregation. Notably, the encapsulated wild-type pneumococci were also surrounded by some vesicle-like structures that provided a rough texture to the bacterial surface, which was predominantly visible in CDM**+** and appeared to contribute to aggregation (**Figure 2**, **Supplementary Figure S1**). Furthermore, serotype 4 strain TIGR4, which is an invasive clinical isolate, formed longer chains in the complex THY medium compared to the minimal media. TIGR4 wild-type strain also displayed pili-like structures on the surface and mostly near the septum region.

To validate the growth differences observed between CDM and CDM+ media, we further determined the number of viable bacteria (CFU/mL) at defined time points during growth. As shown in **Supplementary Figure S5** (Panel B), *S. pneumoniae* EF3030 was assessed at early, mid and late exponential phases. The CFU data confirmed that the elevated OD_600_ obtained in CDM+ medium correspond to a higher number of viable cells, supporting the conclusion that CDM+ enhances bacterial proliferation. However, in CDM, EF3030 did not grow beyond OD_600_ 0.5. We acknowledge that OD_600_ readings can be affected by e.g., cell aggregation, however, the CFU data demonstrated that the increased optical density correlated with bacterial growth. In addition, growth curve analysis of the TIGR4Δ*cps* strain (**Supplementary Figure S5**, Panel A) showed that CDM+ not only leads to a higher bacterial density but also improves growth kinetics by reducing the lag phase and increasing the growth rate. This is in contrast to its slower growth in CDM, further emphasizing the advantages of CDM+ medium. These findings substantiate our conclusion that the optimized CDM+ medium promotes enhanced and more robust growth of pneumococcal strains under nutrient-defined conditions.

### 3.3 Global proteome analysis of *S. pneumoniae* EF3030, serotype 19F

To elucidate the physiological profile of *S. pneumoniae* strain EF3030 at protein level when cultured in the minimal medium CDM+, we conducted a comprehensive proteomic analysis of both the cellular and the supernatant fractions. Although the genome of serotype 19F strain, EF3030 has been published (Junges et al., 2019), a detailed proteome profile is not yet available for this strain nor for any other serotype 19F strain. Our goal was to characterize the protein composition of *S. pneumoniae* strain EF3030 across different growth phases (early exponential, mid-exponential, and late exponential phases) using mass spectrometry.

#### 3.3.1 Experimental setup and protein identification

**Figure 3:**
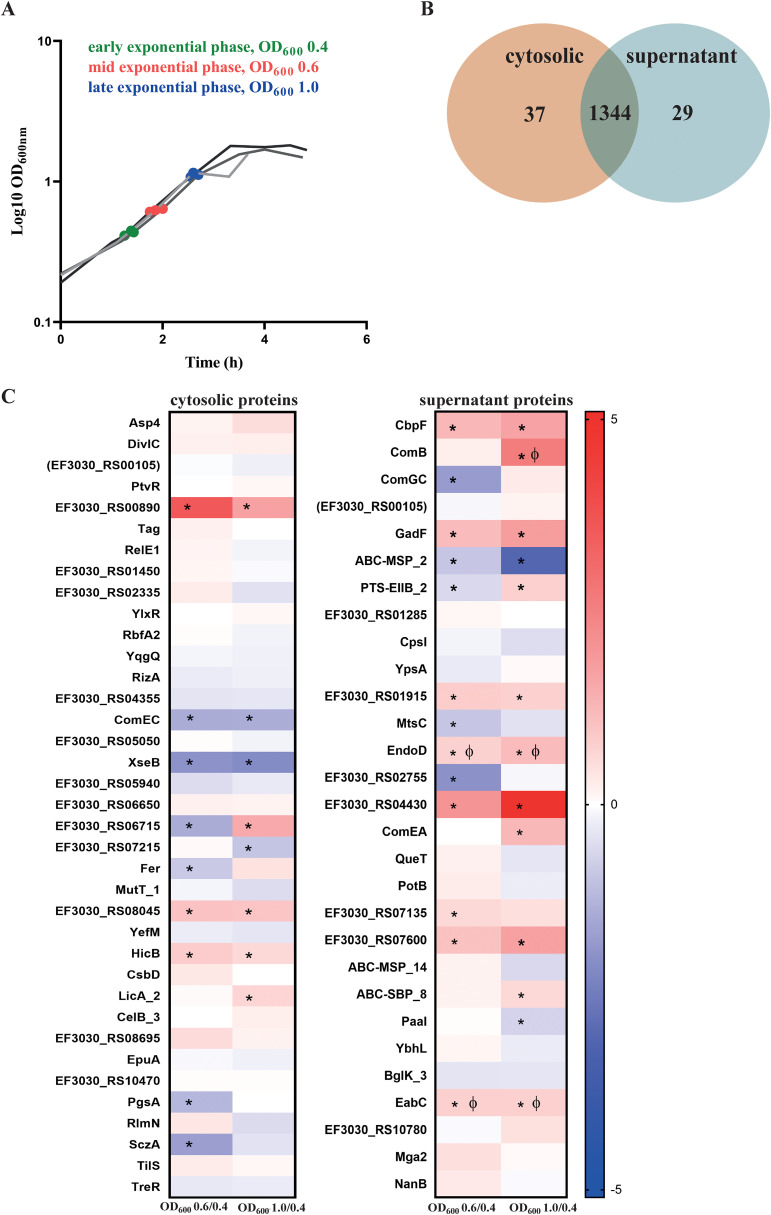
Identification of pneumococcal proteins exclusively detected in the cytosol or supernatant fractions. **(A)** Sampling of bacterial cultures and their corresponding supernatants were done at different exponential growth phases —early (OD_600_ = 0.4, green), mid (OD_600_ = 0.6, red), and late (OD_600_ = 1.0, blue). The three lines represent the biological replicates. **(B)** A total of 1,381 proteins were identified in the cytoplasm and 1,373 proteins were identified in the supernatant, with two or more than two peptides analyzed by mass spectrometry. Of these, 37 proteins were exclusively found in the cytosol, whereas 29 proteins were uniquely present in the supernatant, as depicted in the Venn diagram. **(C)** Abundance of the proteins detected exclusively in the cytosol and the supernatant. Heatmaps represent the signal log ratio comparisons during early, mid and late exponential growth phases. Φ indicates significant values, p-value less than 0.05. * Indicates absolute fold change greater than or equal to 1.5. The protein EF3030_RS00105 is in ‘brackets’ because the identified peptides could originate from other annotated proteins.

*S. pneumoniae* EF3030 was cultured in CDM**+** and harvested at three growth phases, defined by optical density (OD_600nm_) values of 0.4 (early exp), 0.6 (mid-exp), and 1.0 (late exp) (**Figure 3A**). Total protein was isolated from both the cytosolic and supernatant fractions and proteome analysis was performed using the PneumoWiki annotated genome of strain EF3030. NZ_CP035897.1 predicts 1862 protein-coding genes, we were able to successfully identify 1381 proteins in the cytosolic fraction and 1373 proteins in the supernatant fraction, with more than two peptides with ion q-values less than 0.001 (**Figure 3B**). These numbers represent 74.16% and 73.73% of the annotated theoretical proteome in the cytosolic and supernatant fractions, respectively. The high number of different proteins in the supernatant indicated partial cell lysis already at an early stage. A comparison revealed 1,344 proteins common to both fractions, with 37 and 29 proteins uniquely identified in the cytosol and supernatant, respectively (**Figure 3B**).

#### 3.3.2 Proteins identified in the cytosolic fraction

In the cytosolic fraction, 37 proteins were uniquely identified (**Figure 3C**), including key stress response and growth-regulatory proteins. ComEC, which is involved in DNA internalization related to competence and PtvR, important for fitness coping with antibiotic and stress, were detected in the cytosol (Pimentel and Zhang, 2018; Liu et al., 2017). Notably, the toxin-antitoxin system components YefM, RelE1, and HicB, which are implicated in bacterial persistence, were identified in this fraction only. Bacterial persistence is a crucial factor in the survival of *S. pneumoniae* within the host and during antibiotic treatment. HicB was of particular interest, as it exhibited a 1.7-fold increase during the mid-exponential phase and 1.5-fold during the late exponential phase (**Supplementary Excel Table S1**).

#### 3.3.3 Proteins identified in the supernatant fraction

The supernatant fraction contained 29 unique proteins (**Figure 3B**, **Supplementary Excel Table S2**). Most of these proteins were truly secreted or surface associated, including CbpF, ComGC, ComB, ComEA, NanB, EndoD, MtsC, QueT, CpsI, and EabC (**Figure 3C**). However, we also detected some cytoplasmic proteins such as Mga2, a virulence factor transcriptional regulator, together with Paal, and YbhL, mainly due to a substantial lysis rate. Notably, a significant proportion of the detected proteins in the supernatant fraction contribute directly or indirectly to virulence, including NanB, CpsI, ComEA and EndoD. These proteins exhibited distinct abundance patterns during growth, with EndoD and ComEA increasing in later growth phases, whereas CpsI showed a decrease in abundance under the same conditions. Furthermore, EabC, associated with a domain that could be linked to host interaction and the competence factor ComB displayed significant increases in abundance during the late exponential phase. The choline-binding protein CbpF also displayed a notable 3.2-fold increase during the late log phase. The surface proteins, including CpsI and ComEA, demonstrated phase-dependent abundance fluctuations, reflecting the adaptive response of *S. pneumoniae* to changing growth conditions.

#### 3.3.4 Protein composition comparison: cytosol vs. supernatant

The comparative analysis of the relative iBAQ values for cytosolic proteins and proteins present in the supernatant revealed a wide range of protein abundances, spanning approximately 6 log10 steps (**Supplementary Figure S3A**). Highly abundant proteins, including Gap (GAPDH), Tuf, enolase (Eno), EF3030_RS05545 (PtsH), EF3030_RS02365 (Pgk) and ribosomal proteins (e.g., RplX), were found in both fractions. Notably, when the total proteins detected in the late exponential phase were analyzed, LytN and SpxB were found among the top 10 highest abundant proteins. LytN, a murein hydrolase, plays a crucial role in cell division and cell wall integrity. Overexpression of LytN has been shown to cause cell lysis in other bacteria like *S. aureus* (Frankel et al., 2011). SpxB, a pyruvate oxidase, is key enzyme for H_2_O_2_ production (Bättig and Mühlemann, 2008). The protein composition in the supernatant fraction in general mirrored that of the cytosolic profile (**Supplementary Figure S3B**). Proteins that were at least 100-fold enriched in the supernatant fraction, including those involved in cell surface attachment and extracellular functions, such as PfbA, ZmpD, ZmpB, MapP, EF3030_RS10100, EF3030_RS00555, and PcsB. Conversely, we identified the ribosomal protein RpmC, EF3030_RS06250 (AroH), EF3030_RS02670, EF3030_RS02470, and EF3030_RS10365 as being at least 20-fold enriched in the cytosolic fraction.

**Figure 4:**
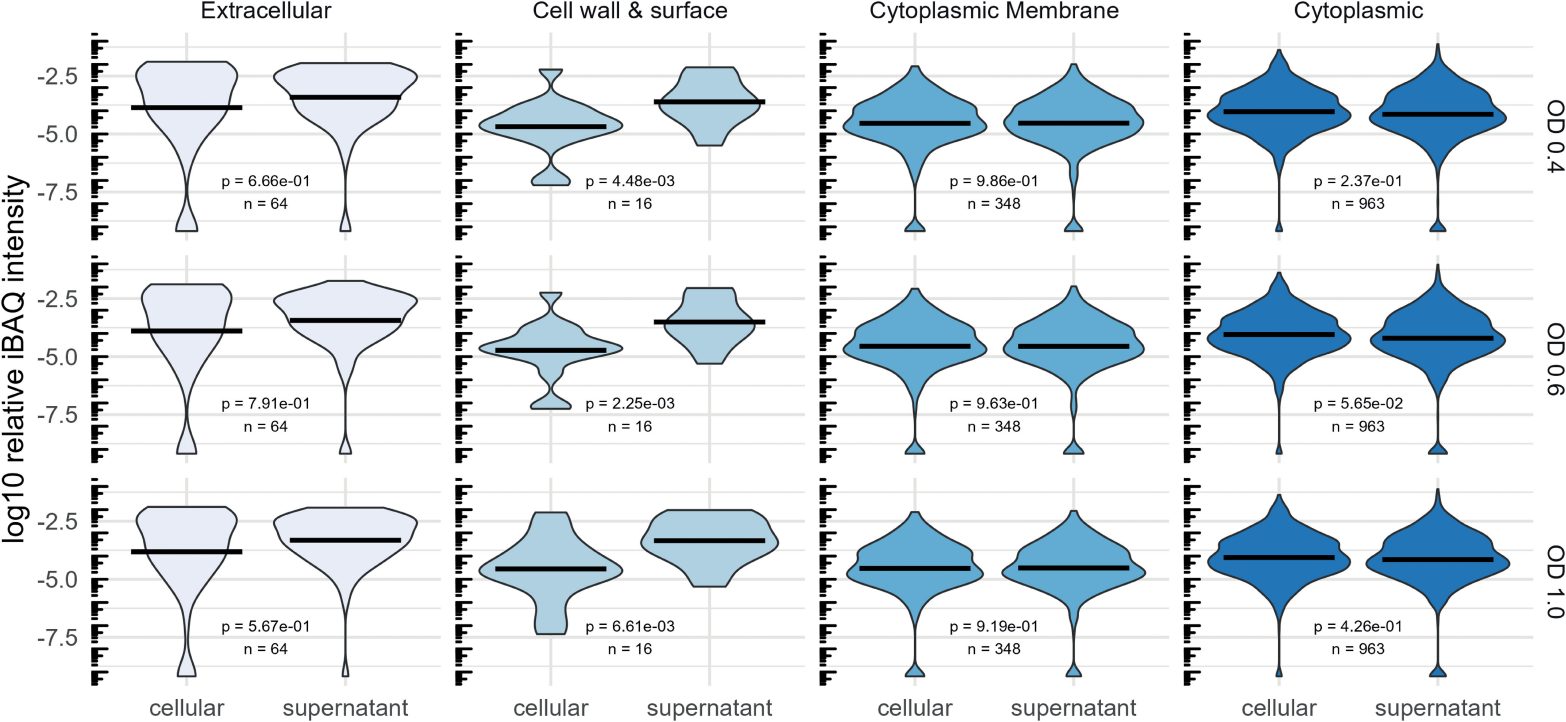
Comparison of the abundance of proteins regarding their predicted localization by DeepLocPro (Moreno et al., 2024). The violin plot shows log10-transformed relative iBAQ values. Medians are indicated as lines. Significant differences of mean relative abundances between the cellular and supernatant (secretome) fraction was tested using a Wilcoxon-test.

*In silico* predictions of protein localization by DeepLocPro (Moreno et al., 2024) indicate that the supernatant fraction is substantially enriched in extracellular proteins and cell wall and surface proteins across all analyzed growth states (**Figure 4**). Notably, the relative abundance of membrane and cytoplasmic proteins does not differ between the two fractions, a finding that is consistent with the high frequency of lysis of pneumococcal cells (Terrasse et al., 2015; Eldholm et al., 2009)

#### 3.3.5 Growth-dependent changes of the cellular proteome and of the exoproteome

**Figure 5:**
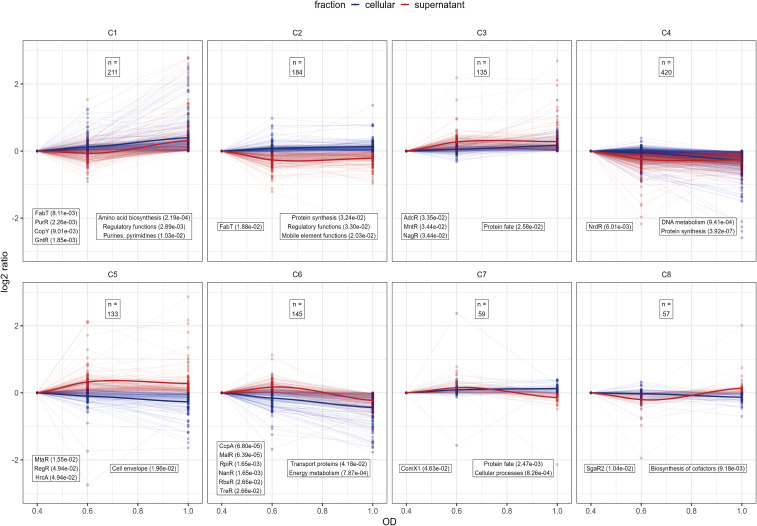
Analysis of protein profile clusters. For clustering the log2 protein intensity ratios compared to OD_600nm_ 0.4 were used for the supernatant and cellular fraction. Line plots of protein profiles for each cluster are shown, with cellular fraction profiles are depicted in blue and supernatant fraction profiles in red. To show the general cluster profile the LOESS-fit of all protein profiles are depicted as solid line (Jacoby, 2000). The total number of proteins in each cluster is indicated at the top of each cluster panel. Each cluster was tested for over-representation of proteins belonging to known regulons and known TIGRFAM functional annotations according to PneumoWiki (https://pneumowiki.med.uni-greifswald.de). Over-representation was assessed using one-sided Fisher’s exact test (p-value ≤ 0.05). Over-represented regulons and functional categories are depicted in the left box and right box, respectively, along with their corresponding p-values in brackets.

To investigate pneumococcal physiology in the newly developed minimal medium CDM**+**, pneumococcal cells and supernatants were collected at early, mid, and late exponential growth phases as shown in **Figure 3A**. Comparative analysis of the mid and late exponential phase samples versus early exponential phase samples allowed for the examination of changes in protein expression profiles, metabolic alterations, and bacterial cell adaptations across varying growth states and cell densities (**Figure 5**). This information is crucial for the development of therapeutics targeting bacterial proteins, as it can provide insight into strategies to inhibit bacterial growth and progression of infection at different stages. The distinct growth phases correspond to different numbers of bacteria during colonization or dissemination in the blood or cerebrospinal fluid. Initially low bacterial number initiate adherence or cross host barriers and subsequent accumulation or biofilm formation contribute to the progression of infection (Hall-Stoodley et al., 2004).

To gain a general overview about the protein abundance changes along the growth states, the obtained proteome profiles of *S. pneumoniae* EF3030 were normalized to the protein abundance at the early exponential phase and then clustered. Hierarchical clustering was applied (**Supplementary Excel Table S3** for the data) and clusters were defined by cutting of 40% of the total tree distance. This resulted in eight general protein clusters representing the proteome dynamics along the growth states in the supernatant and cytoplasmic fraction (**Figure 5**). Clusters C1 and C6 represent proteins associated with metabolic functions as identified using overrepresentation analysis of known regulons and functional TIGRFAM categories. Of note, proteins belonging to the fatty acid biosynthesis FabT regulon and the purine synthesis PurR regulon as well as amino acid biosynthesis proteins tend to accumulate along the growth state in the cytoplasmic and supernatant fraction, whereas proteins belonging to the central sugar metabolism CcpA regulon, the maltose utilization MalR regulon, sialic acid utilization NanR and RpiR regulons, fructooligosaccharides utilization RbsR regulon and the trehalose utilization TreR regulon decreased in abundance in the cytosolic and supernatant fraction during progression through growth phases (**Figure 5**). In that sense, our data reveal a shift from sugar-based glycolysis to other metabolic aspects over time when growth in the newly optimized medium and metabolic dynamics also shape the pathophysiological potential of pneumococci. In general, the CcpA regulated central metabolism is closely linked to virulence (Iyer et al., 2005; Carvalho et al., 2011; Zhang et al., 2023) and CodY-driven metabolic aspects are essential in colonization and infection processes of pneumococci (Hendriksen et al., 2007; Johnston et al., 2015). Proteins of the clusters C3 and C5 could be quantified in higher abundance in the later phases of growth in the supernatant. These clusters comprised of the zinc acquisition regulator AdcR, manganese homeostasis MntR regulon, the N-acetylglucosamine utilization and the hyaluronate utilization, RegR regulon as well as protein’s function associated to the cell envelope, demonstrating the need of metal ions and accumulation of classic extracellular proteins during later growth phases. Proteins of the DNA metabolism, protein biosynthesis (cluster C4) and biosynthesis of cofactors (cluster C8) remained relatively stable during the exponential growth phase, highlighting their essential role in the bacterial physiology.

**Figure 6:**
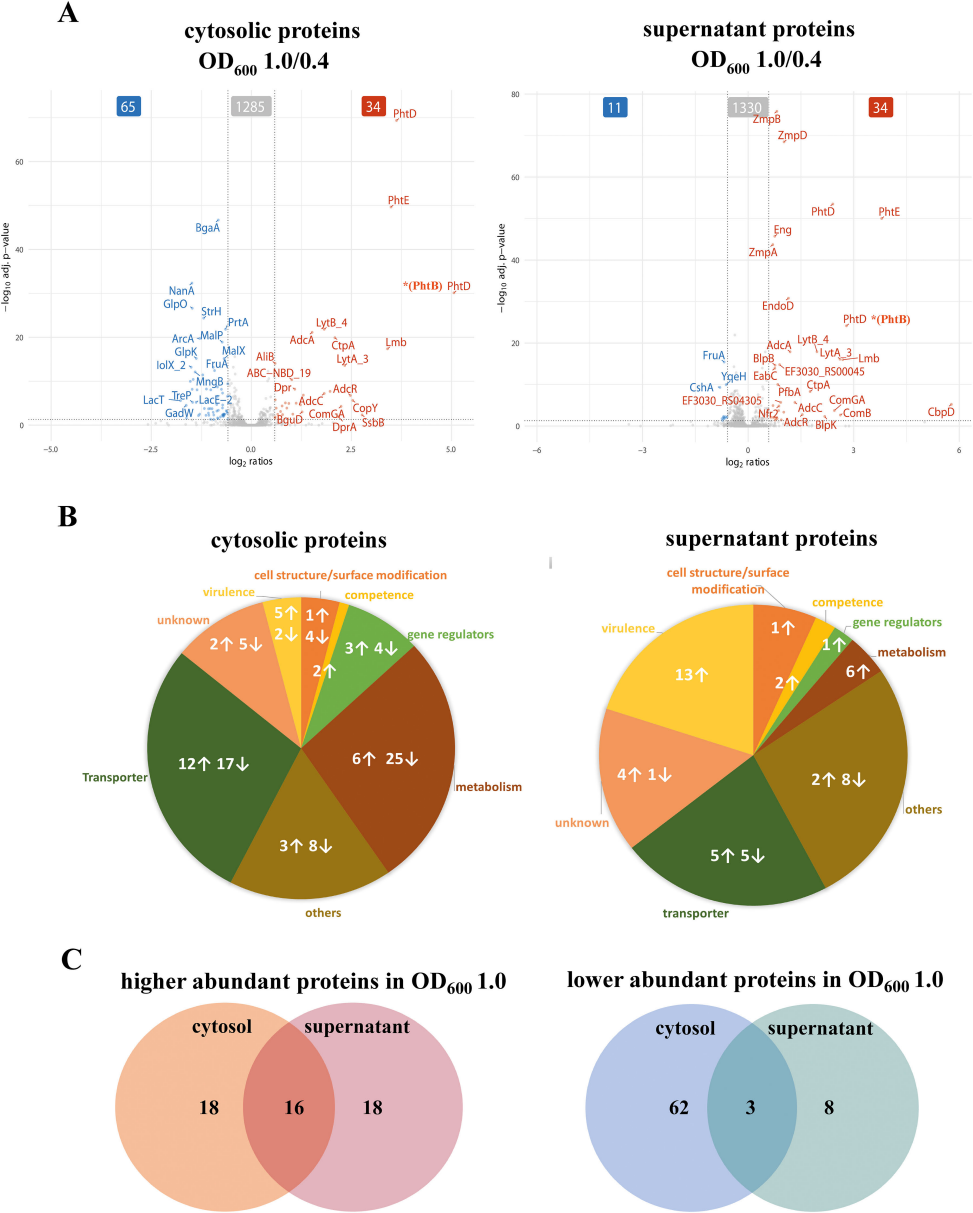
Quantification of the abundant proteins in the cytosol and the supernatant fractions during exponential growth. **(A)** Significant changes in protein abundance during the late exponential phase compared to the early exponential phase are visualized using volcano plots. The data represents the significant signal log ratio comparison between the different optical densities, with p-value less than 0.05 and a fold change greater than 1.5-fold. Proteins with higher abundance are labelled red, whereas proteins with a decrease in protein abundance are marked in blue. **(B)** Pie charts illustrate the significantly abundant proteins and their functions in the cytosol and supernatant during the exponential growth phase. Arrows indicate an increase or a decrease in protein abundance by comparing late log to early exponential phase. **(C)** A selection of proteins with increased abundance in the cytosol or supernatant, as well as proteins with reduced abundance in the cytosol or supernatant, at late growth.

To gain detailed insights into the growth state dependent changes in protein levels in both the cytosolic and supernatant fraction, pairwise comparisons were performed for the samples harvested in the late exponential phase to samples harvested in early exponential phase using the ROPECA approach (Suomi and Elo, 2017) (**Figure 6**, **Supplementary Excel Tables S1** and **S2**). Only proteins with an absolute fold change of 1.5-fold or greater and an adjusted p-value less than 0.05 were considered as significantly altered and were analyzed (**Supplementary Table S4**). 34 proteins showed increased abundance in both fractions, the cytosol and supernatant. In contrast, 65 proteins were significantly lower in abundance in the cytosol fraction and 11 proteins were significantly lower in the supernatant fraction (**Figure 6A**). We then classified these differentially expressed proteins into functional groups of interest: transporter proteins (29 in cytosol and 10 in supernatant), gene regulation proteins (7 in cytosol and 1 identified in supernatant), metabolic proteins (31 in cytosol and 6 in supernatant), virulence and competence factors (9 in cytosol and 15 in supernatant), proteins with others functions (11 in cytosol and 10 in supernatant) and proteins of unknown function (7 in cytosol and 5 in supernatant) (**Figure 6B**). Detailed description along with the fold change is shown in the **Supplementary Tables S4A, B**.

To determine the overlap of the proteins shown in the volcano plot (**Figure 6A**) in both the cytosolic and the supernatant fraction, a Venn diagram (**Figure 6C**) was used. Sixteen proteins (the Pht family proteins, the Adc system proteins involved in Zinc homeostasis, ComGA, sortase SrtA, YdcP_2, BguA, Mip, Nfr1, Nfr2) were found to significantly increased in both the cytosol and the supernatant, while three proteins (FruA, TreP and GadW) were significantly reduced in both fractions (**Supplementary Tables S4A, B**, proteins marked with “*”).

#### 3.3.6 Transporters

During the late-log phase (OD_600_ 1.0), increased abundance of various transporter proteins was measured in both the cytosolic and supernatant fractions (**Figure 6B**, **Supplementary Table S4A**). Notably, PTS and ABC transporters involved in sugar uptake, such as BguB, BguC, and BguD, were more abundant in the cytosol. Moreover, zinc and metal transporters, such as AdcA, AdcAII, AdcC, and CtpA, were elevated in both fractions, indicating the bacterial adaptation to metal ion availability during growth, as indicated by overrepresentation of the AdcR regulon in cluster C3 (**Figure 5**). In contrast, several sugar transporters, including MalC, BrnQ, GadW, FruA, and TreP exhibited reduced abundance during the late-exponential phase (**Supplementary Table S4B**). This is in line with the observation that the general sugar metabolism decreases in later growth states (cluster C6; **Figure 5**).

#### 3.3.7 Gene regulators

Several transcriptional regulators, including the Zn-dependent regulator AdcR showed increased abundance in both the cytosolic fraction and the supernatant fraction during the late-exponential phase (**Figure 6A**, **Supplementary Table S4A**). Other regulators like GntR and CopY were significantly increased in the cytosolic fraction during the later growth phase (**Supplementary Excel Table S1**). In contrast, other regulators such as NmlR and FruR, exhibited a significant reduction in the cytosol and were also reduced in the supernatant during later growth phases suggesting growth-phase-specific regulation of bacterial adaptation and stress response (**Supplementary Table S4B**, **Excel Tables S1, S2**).

#### 3.3.8 Metabolic proteins

Metabolic proteins involved in amino acid and sugar metabolism, such as galactokinase (GalK), LacG, and arginine deaminase system (ArcA, ArgF (ArcB), ArcC), exhibited decreased abundance during the late-log phase in the cytosol (**Supplementary Figure S2**, **Supplementary Table S4B**). In contrast, some metabolic enzymes, including GuaA2, PEP phosphomutase, YdpC_2, and BguA, showed increased abundance in the cytosol, indicating changes in metabolic activity during different growth phases (**Supplementary Table S4A**). This metabolic adaptation is also reflected by the identification of the two general metabolic clusters C1 and C6 (**Figure 5**). In general, sugar metabolism proteins tend to decrease during the late exponential phase in both the cytosolic and supernatant fractions, whereas the abundance of proteins involved in amino acid and purine and pyrimidine biosynthesis increased.

This shift in metabolic regulation is further supported by gene set enrichment analysis (GSEA), as depicted in **Supplementary Figure S2**. The plot illustrates functional annotation and regulon-based enrichment across different growth phases (OD 0.6/0.4 and OD 1.0/0.4). The color of the dots represents the direction of normalized enrichment scores (NES), with red indicating positive enrichment and blue indicating negative enrichment. The size of each dot corresponds to the absolute NES value, highlighting the magnitude of enrichment. Notably, during the transition from mid to late exponential phase, cellular processes and nucleotide metabolism were positively enriched, while energy metabolism showed a negative enrichment. Additionally, the CcpA regulon, known for its role in carbon metabolism, exhibited significant negative enrichment, reinforcing the observed decline in sugar metabolism proteins (**Supplementary Figure S2**). These findings collectively suggest a metabolic shift favoring nucleotide and amino acid biosynthesis over sugar metabolism during later growth stages.

#### 3.3.9 Competence and Other proteins

Remarkably, we observed a more than 2-fold increase of the competence type IV pilus ATPase ComGA in both pneumococcal fractions during the late exponential phase (**Figure 6A**, **Supplementary Table S4A**). In addition, ComB, a component of the competence system, was found to be 6.1-fold increase in the supernatant at the late exponential phase (**Figure 6A**, **Supplementary Table S4A**). Competence requires a complex and well-timed interplay of several regulons (such as ComE, ComX, BlpR) (Winkler and Morrison, 2019; Weyder et al., 2018) We found that the ComX1 regulon has a general peak in the mid exponential phase while protein levels decrease in the supernatant fraction during the late exponential phase (cluster C7; **Figure 5**)

Additionally, several cytoplasmic proteins involved in DNA processing and repair showed increased abundance over time (**Figure 6A**, **Supplementary Table S4A**). The single-strand DNA-binding protein, SsbB (EF3030_RS09295) stabilizes single-stranded DNA during replication, recombination, and repair. DprA facilitates natural transformation by binding and guiding incoming DNA into the chromosome. Sortase A, although primarily responsible for anchoring surface proteins, may support competence by attaching transformation-related proteins to the cell surface. SsbB and DprA are directly involved in DNA uptake and processing during natural transformation, a process that enhances genetic adaptability. Sortase A might contribute to this process by supporting the display of competence-associated factors on the cell surface. In the supernatant fraction, bacteriocin (EF3030_RS00555) or bacteriocin-like protein BlpU, bacteriocin secretion accessory protein (EF3030_RS02515), serine hydrolase (EF3030_RS00045), a glycoside hydrolase (EF3030_RS10670), and a surface protein (EF3030_RS05660) increased significantly at late exponential phase (**Supplementary Table S4A**). The secreted proteins (bacteriocins, hydrolases, and surface proteins) indicate enhanced bacterial competition, environmental adaptation, and potential host interactions. In contrast, out of the 11 significantly reduced proteins in the supernatant (**Figure 6C**), 8 proteins were exclusively found in the supernatant including YqeH, RsgA, ParB partition protein, methyltransferase domain containing protein, and the DEAD box helicase, whereas 3 were also found in the cytosol (**Figure 6C**, **Supplementary Table S4B**).

#### 3.3.10 Protein profiles of surface proteins and proteases - investigation of the repertoire of selected virulence factors

**Figure 7:**
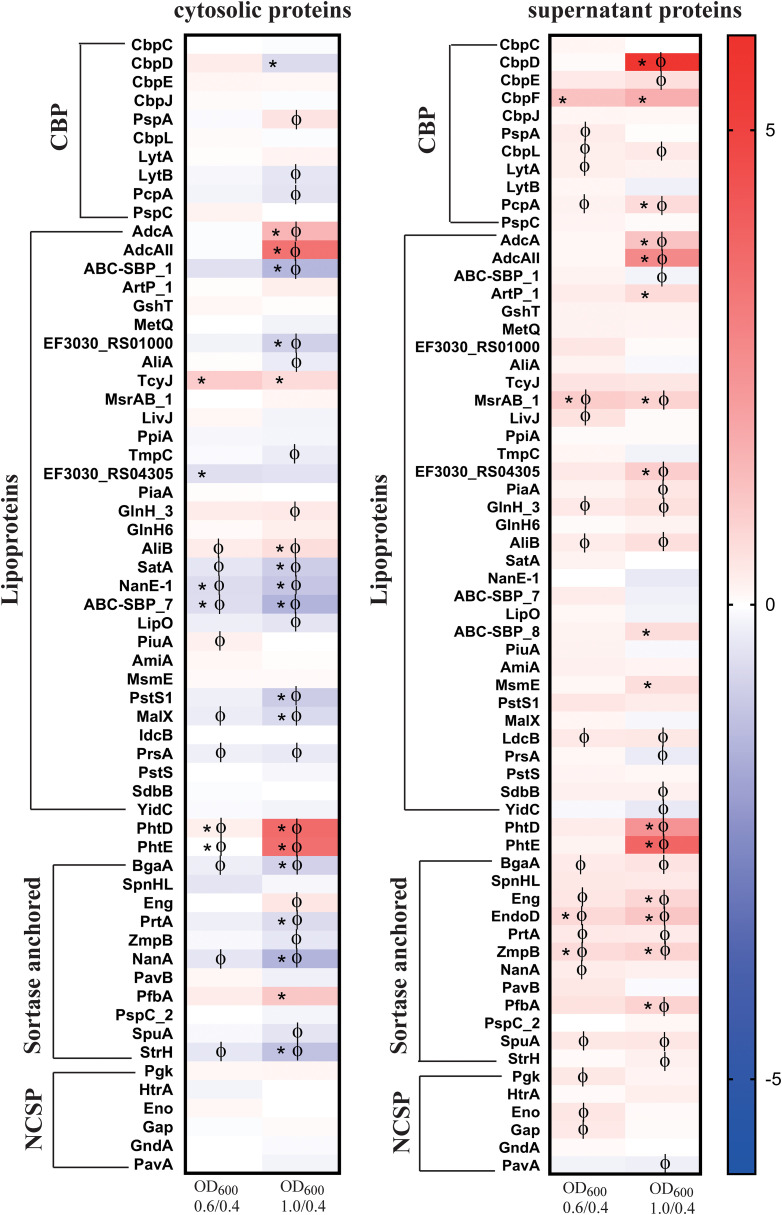
Heat maps of surface associated proteins with two or more peptides identified both in the cytosol and the supernatant of strain EF3030 by mass spectrometry analysis. The abundance of detected proteins was classified as choline-binding proteins (CBP), lipoproteins, sortase-anchored proteins and non-classical surface proteins (NCSP) in the cytosol and supernatant. The heat map represents the signal log ratio values between mid-exponential phase to early exponential phase and late exponential phase to early exponential phase. Φ indicates significant values, p-value less than 0.05. * Indicates an absolute fold change greater than or equal to 1.5.

For comparative analysis, we classified various proteins based on their surface anchoring mechanisms (**Figure 7**) (Lane et al., 2022; Bergmann and Hammerschmidt, 2006; Pérez-Dorado et al., 2012). The data revealed a significant increase in the signal log ratio of several lipoproteins, choline-binding proteins, sortase-anchored proteins, and non-classical surface proteins (NCSP) in both the cytosolic and supernatant fractions comparing the mid and late log phase to the early log phase (**Figure 7**, **Supplementary Excel Tables S1, S2**). Choline-binding proteins were detected in both the cytoplasmic and supernatant fractions, with the most striking increase observed for the murein hydrolase CbpD, which exhibited a 54.7-fold increase in abundance in the supernatant during the late log phase (**Figure 7**, **Supplementary Table S4A**). A 3.2-fold increase in abundance was also detected for CbpF, which was exclusively found in the supernatant (**Figure 3**). In contrast, while all choline-binding proteins were present in the cytoplasmic fraction at low levels, their abundance remained relatively unchanged throughout growth. We observed PhtD to be the most significantly elevated protein detected in both the cytoplasmic and supernatant fractions during the later stages of log growth (**Figure 6A**). PhtD and PhtE are members of the histidine triad protein (Pht) family, characterized by four to six histidine motifs that bind divalent metal cations, such as Zn^2+^ (Bersch et al., 2013).

Among the lipoproteins, AdcA and AdcAII were the prominent proteins with higher abundance, found in the late log phase in both the cytosolic fraction and extracellular fraction. A significant increase of ABC_SBP_8 was observed at the late exponential phase in the extracellular fraction, whereas other ABC-SBP (-SBP_1 and –SBP_7) components remained unchanged throughout growth. Furthermore, MsmE, ArtP_1, RS04305, and AliB as subunit of the Ami oligopeptide ABC transporter showed higher abundance in the late log phase. AliA, another subunit of the Ami transporter system, did not exhibit significant changes in abundance during growth. Notably, MsrAB, as part of the detoxification system for oxidized proteins, was consistently detected in higher abundance across all growth conditions compared to the early exponential phase. Additionally, several other lipoproteins like LdcB or SdbB remained stable during growth (**Figure 7**).

Significant differences in the abundance of sortase-anchored proteins compared to the early exponential phase were observed in the supernatant fraction. A notable increase in the abundance of EndoD, which contains an Ig-like domain, was found in the extracellular fraction. Additionally, increases were also observed for Eng, ZmpB, and PfbA. In contrast, the neuraminidase NanA level remained unchanged during growth, and similarly, SpnHL SpuA, StrH and PspC_2 did not exhibit alterations. A decreased abundance was detected for the adhesion PavB in the late exponential growth phase. Furthermore, decreases in NanA and StrH levels were observed in the cytoplasmic fraction during exponential growth phases (**Figure 7**).

The non-classical surface proteins (NCSP) like enolase (Eno) and GAPDH (Gap) were found in abundance in both the cytosol and the supernatant (**Supplementary Table S4A**). They were observed in the early exponential phase and significantly increased during the mid-exponential phase (**Figure 7**). Notably, PavA (pneumococcal adherence and virulence factor) lacking a secretion signal and domains for cell surface anchoring increased significantly during the late exponential phase in the supernatant fraction.

**Figure 8:**
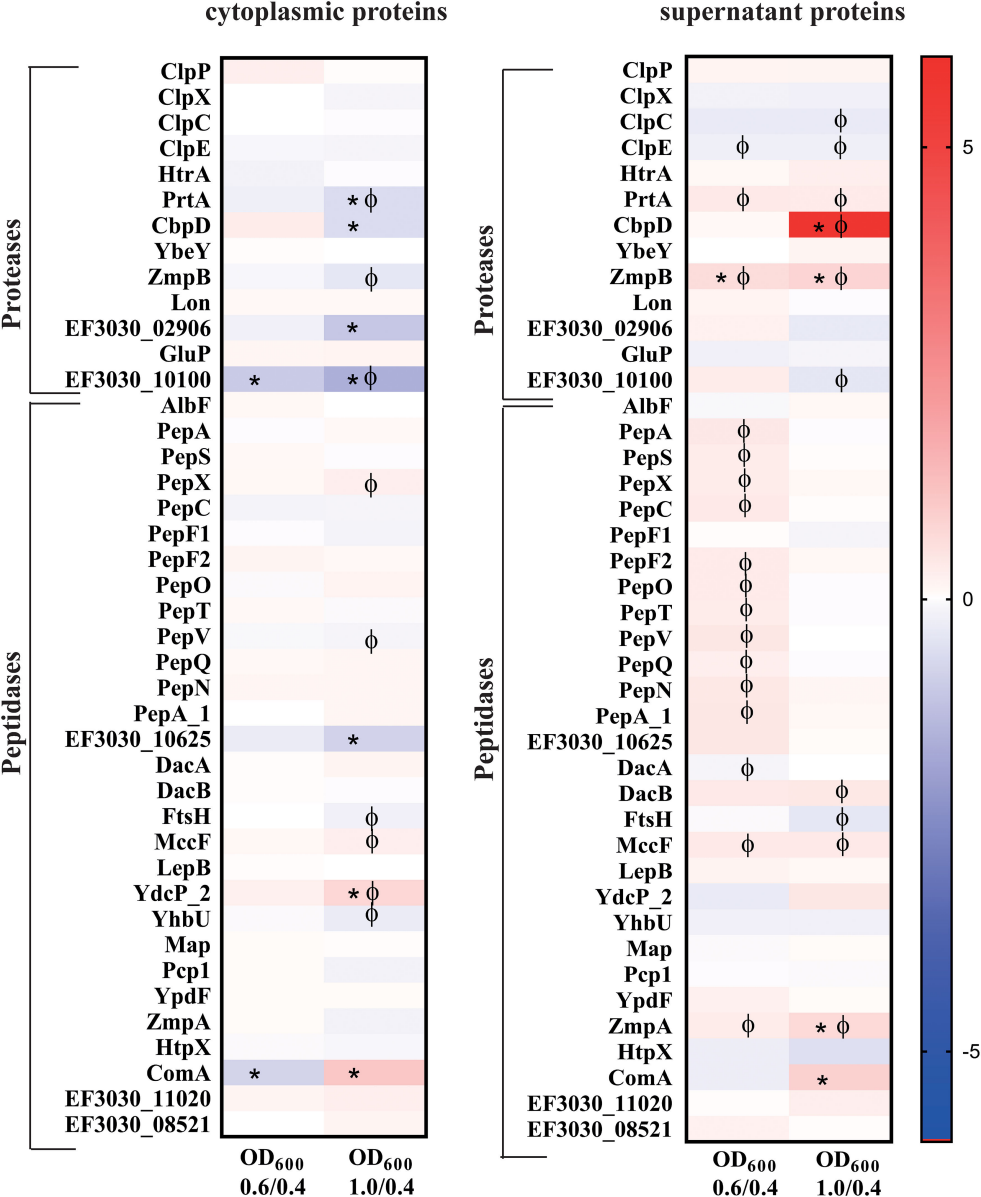
Heat maps illustrating a selection of proteases and peptidases with two or more peptides detected both in the cytosol and the supernatant of strain EF3030 by mass spectrometry analysis. The heat maps represent the signal log ratio values between mid-exponential phase to early exponential phase and late exponential phase to early exponential phase. Φ indicates significant values, p-value less than 0.05. * Indicates an absolute fold change greater than or equal to 1.5.

Proteases and peptidases are essential for multiple physiological processes in bacteria. Most of the proteases and peptidases discussed in this study (**Figure 8**) maintains important cellular functions attributing to survival, adaptability and pathogenesis (Marquart, 2021). The typical Clp protease components (ClpP, ClpX, ClpC and ClpE) and the chaperone ClpL, were found in both fractions. The ClpP ratio decreased in the cytoplasmatic fraction during the late exponential phase compared to the early growth phase, however there is no significant change in either fraction during the exponential growth. Interestingly, out of the three extracellular serine proteases present in the colonizing strain, EF3030, the secreted serine proteases PrtA and HtrA were identified in both the fractions. No significant change of HtrA was observed during the exponential growth. In the cytosol, PrtA significantly reduced more than 1.5-fold during the late exponential phase, however it increased significantly in the supernatant during the exponential growth (**Supplementary Table S4A**). CbpG, the third serine protease was not detected under our experimental conditions. YdcP_2, a member of the U32 family peptidases, showed an increased abundance during growth in both fractions.

Among the pneumococcal peptidases, PepX and PepO exhibited increased abundance during late exponential phase in the cytoplasmic fraction compared to the early exponential phase, whereas PepS and AlbF showed a decrease in abundance during growth. Most of these peptidases had a decreased abundance during exponential growth in the extracellular fraction. AlbF was an exception, with a small increase in abundance observed at the late exponential phase.

## 4 Discussion

Understanding the dynamics of protein production during pneumococcal growth, colonization, or invasive disease is essential for elucidating the molecular mechanisms underlying the pathophysiology of these pathobionts. This knowledge is important for identifying vaccine candidates and defining new targets for therapeutic interventions as well as developing sophisticated diagnostic tools. Our study employed an optimized minimal medium (CDM+) mimicking a nutrient-limited host compartment, such as the URT, to characterize the proteomic landscape of the colonizing serotype 19F strain EF3030.

To cultivate the pneumococcal strains under nasopharynx-mimicking conditions, a chemically defined medium (CDM) based on RPMI 1640 was initially formulated that included components such as bicarbonate, glutamine, glycine, choline chloride, and adenine-uracil (Schulz et al., 2014). The nasopharyngeal environment is characterized by low CO_2_ levels; therefore, bicarbonate was added in the minimal medium CDM, which provides an alternative source of hydrogen carbonate (HCO_3_^−^). This was particularly relevant given the findings of Burghout and colleagues, which highlighted the importance of carbonic anhydrase (PCA) activity in pneumococcal adaptation to CO_2_-poor conditions (Burghout et al., 2013). Carbonic anhydrase facilitates the reversible conversion of CO_2_ to bicarbonate, which is essential for fatty acid biosynthesis and polyglutamyl folate biosynthesis. No significant changes in carbonic anhydrase (MtcA1) levels were observed in our presented dataset, reinforcing the idea that it was not required due to the bicarbonate supplementation in the minimal medium. Additionally, several proteins from the *fab* gene cluster involved in fatty acid biosynthesis, such as FabD and FabG, showed significantly higher abundance in both the cytosolic fraction and the supernatant during the late exponential phase of our study. Furthermore, the increased presence of FabK and FabZ in the supernatant during the late logarithmic phase suggests that the bacteria are directly utilizing bicarbonate to support growth and fatty acid synthesis. Additionally, we included glutamine in CDM because *S. pneumoniae* is auxotrophic for glutamine (Gln) (Härtel et al., 2011). Glutamine, taken up by a glutamine ABC transporter system, is crucial for pneumococcal metabolism and serves as a precursor for amino acids and nucleotides. Deprivation of glutamine has been shown to impair bacterial growth and virulence (Härtel et al., 2011). Uracil, another component of CDM was included because it has been previously identified as a limiting factor for pneumococcal growth (Carvalho et al., 2013).

To further optimize the CDM medium and growth of streptococcal and pneumococcal strains, we developed CDM+ by supplementing CDM with iron, manganese, and methionine. *S. pneumoniae* has a relatively low iron requirement, as it lacks a respiratory chain and does not contain cytochromes (Lanie et al., 2007). Nevertheless, the bacterium still requires iron for enzymes that contain iron-sulfur (FeS) clusters, such as anaerobic ribonucleotide reductase (Lanie et al., 2007). Previous studies have shown that *S. pneumoniae* strain TIGR4 exhibits reduced growth under iron-depleted conditions, which can be restored with iron supplementation (Gupta et al., 2009). In low-iron environments, pneumococci form long chains, indicative of impaired cell separation. Furthermore, mutations in the genes encoding the ABC transporters responsible for iron acquisition (PiaABCD and PiuBCDA) attenuated the virulence of *S. pneumoniae* in a mouse infection model (Brown et al., 2001, 2002).

Interestingly, in the present study, the strains, EF3030, TIGR4 and D39 exhibited clumping in CDM**+**, suggesting that the presence of iron may be a contributing factor. Previous studies also showed that elevated hemoglobin levels induce pneumococcal clustering and the secretion of factors that promote cell aggregation, suggesting adhesion and biofilm formation (Gupta et al., 2009).

Manganese was included in CDM+ as it is also an essential element in most organisms, serving as a cofactor for many enzymes involved in phosphorylation, hydrolysis, carbon metabolism, decarboxylation, and oxidative stress (Eijkelkamp et al., 2014). Pneumococcal proteins involved in manganese acquisition, such as MntE, NrdEF, SodD, PsaA, PsaR, and PhpP have a critical role in maintaining manganese homeostasis. When manganese levels are excessive, *S. pneumoniae* employs mechanism to cope with the situation by reducing Mn uptake via the PsaABC transporter system, increasing Mn efflux (MntE), and inhibiting the Fe transport system (Martin et al., 2017). Our proteome data identified MntE, PsaA, MntR and PhpP in both cytosol and the supernatant supporting the role of manganese in cellular processes during growth in CDM+.

Building on this optimized medium, we focused our analysis on the protein profiles of *S. pneumoniae* EF3030 across three distinct growth phases: early (OD_600_ 0.4), mid (OD_600_ 0.6), and late exponential phase (OD_600_ 1.0). Protein abundance was systematically assessed in both the cytoplasmic and culture supernatant fractions, providing a comprehensive view of the dynamic changes in protein abundance as the bacteria transitioned through different stages of growth. In addition, we compared the overall changes in proteome profiles in pneumococcal strains D39V, TIGR4Δ*cps*, and EF3030 cultured in CDM+ to assess the applicability of our findings across different strains. D39V is a serotype 2 strain commonly used in virulence studies and experiments elucidating host-pathogen interaction. Similarly, TIGR4Δ*cps* (capsule mutant of serotype 4) is also known for its high virulence under *in vivo* conditions and used in particular to investigate the impact of virulence factors for pneumococcal pneumonia but also meningitis. Although all strains were cultivated in the CDM+ medium, however, it is important to mention, that medium batches, scientists as well as the analysis strategies of the proteome data slightly differed. Proteomic data of TIGR4Δ*cps* was obtained from an in-house pilot-study (unpublished data) and proteomic data of D39V was obtained from the PRIDE Project PXD061622. The general changes in the proteome profiles of later growth phases were compared to the early exponential growth phase. EF3030 and TIGR4Δ*cps* were compared at OD_600_ 1.0 to OD_600_ 0.4 and the strain D39V was compared at OD_600_ 1.5 to OD_600_ 0.5. We used a GSEA analysis of the known regulons in *S. pneumoniae* as shown in the cluster plot in **Figure 5**. We observed mainly changes in protein abundances of the regulons associated with metabolism in all three strains such as the sugar metabolism regulons CcpA and MalR which (**Supplementary Figure S6**) were higher in abundance during early stages of growth. We observe a much lower abundance of CcpA in EF3030 compared to the invasive strains during the late exponential phase. The invasive strains, but with more pronounced results especially the strain D39V, showed a general decrease of protein abundance of the CodY and FabT regulon compared to EF3030. This indicates metabolic differences between invasive and non-invasive strains. However, there seem to be also differences in the regulons of TIGR4 and D39V, which have already been identified earlier, when analyzing the arginine deiminase system and its regulation in TIGR4 and D39 (Schulz et al., 2014). In addition, D39V showed a decrease of competence-related proteins (ComX1, ComE regulon) in the later growth phase. This is consistent with competence development during the initial exponential growth phase (Zhang et al., 2023). Furthermore, the three strains showed differences between the accumulation of zinc scavenging systems during the later growth phase compared to the early growth phase. The major regulators were compared between these strains to have an insight regarding strain specific differences. Further an extended comparative analysis of the non-invasive and invasive strains would be valuable as a proteogenomic study on pangenome level.

From the EF3030 proteome data, notably, surface-associated and secreted proteins, which are crucial for pathogen-host interactions, exhibited significant variations in abundance depending on the growth phase. These proteins, including choline-binding proteins (CBPs), sortase-anchored proteins, and lipoproteins, are directly involved in bacterial adhesion, invasion, or immune evasion mechanisms and contribute to pneumococcal fitness. As such, they represent promising candidates for the development of vaccines and therapeutic strategies (Morsczeck et al., 2008; Rigden et al., 2003).

We identified over 1344 proteins that are common to both the supernatant and cellular fraction, suggesting a high rate of pneumococcal lysis in the medium already at the early stage of growth. This could be attributed to CbpD, a murein hydrolase, which showed a remarkable 54-fold increase in abundance in the extracellular fraction during the late exponential phase. However, even at lower levels early on, it may still trigger lysis in a subset of cells, potentially initiating a cascade effect. CbpD causes lysis on its own and promotes an activation on LytA and LytC (Kausmally et al., 2005; Eldholm et al., 2009). The abundance of LytA increased in both the cytosol and the supernatant during exponential growth, although this increase was not statistically significant. In contrast, LytC was not detectable in our experimental conditions. We hypothesize that CbpD lyses non-competent sister cells, a process that is referred to as allolysis (Guiral et al., 2005), and the LytA from these cells promotes in-trans lysis of competent pneumococcal cells.

As *S. pneumoniae* colonizes the nasopharynx, hydrogen peroxide (H_2_O_2_) production is thought to play a role in inhibiting competing microbes. SpxB, a pyruvate oxidase and the key enzyme for H_2_O_2_ production, was detected in higher abundance in the supernatant during the mid-exponential phase (OD_600_ 0.6) and reduced during the later growth phase. The early expression of pyruvate oxidase during bacterial growth has also been reported in other studies (Pericone et al., 2000; Lee et al., 2006; Bättig and Mühlemann, 2008) coinciding with the time when pneumococci reach competence (Bättig and Mühlemann, 2008). Previous studies have demonstrated that the deletion of *spxB* abolishes spontaneous transformation, reduces the expression of early competence gene *comC*, and diminishes competence-associated DNA release (Bättig and Mühlemann, 2008). However, the role of SpxB products, H_2_O_2_, in these processes remains unclear. Interestingly, we failed to detect ComC, while we observed a higher abundance of ComB, a protein required for transformation, in the supernatant during the late exponential phases compared to the early exponential phase. Additionally, the late competence-associated proteins ComGA, which are essential for DNA uptake (Balaban et al., 2014), were found in significantly higher abundance both in the supernatant and cytosol, with a nearly 5-fold increase in the supernatant during the late exponential phase compared to both early and mid-exponential phase. Moreover, ComEA, a major component of DNA uptake apparatus for transformation was exclusively found in the supernatant, also increased in abundance during the late exponential phase. A study by Liu and colleagues have demonstrated that the receptor ComEA plays a significant role in the uptake of dsDNA during bacterial transformation, whereas HtrA, a serine protease, specifically degrades ComEA and ComEC to terminate the process (Liu et al., 2019). Other proteins involved in processing internalized single-stranded DNA (ssDNA), such as Dpr, DprA, and SsbB, were also found in higher abundance, although not always statistically significant, in both the cytosol and supernatant during the late exponential phase. *S. pneumoniae* cultivated in the minimal medium retains the ability to take up foreign DNA during the later stages of growth (Claverys et al., 2006). Altogether, these findings provide insights into the timing and regulation of competence and lysis in *S. pneumoniae*, highlighting the dynamic interplay between protein expression, autolysis, and DNA uptake during bacterial growth and colonization. In addition, this demonstrates that the medium allows competence development in *S. pneumoniae* EF3030 during the exponential growth phase.

In our proteomic analysis of *S. pneumoniae* EF3030, we observed a significant increase in pneumolysin abundance (p-value < 0.05) from the early (OD_600_ 0.4) to the late exponential phase (OD_600_ 1.0) in the cytosolic fraction. However, this increase was not significant in the supernatant. In addition to pneumolysin, we identified several immunogenic proteins including PspC, PcpA, PrtA, PcsB, PhtD, PsaA, AliB, PavB, SatA, PnrA, PpmA, PspA, AmiA, LytN, and EF3030_RS10100 in both the cytosol and supernatant during the exponential growth phase. Serological profiling of pneumococcal proteins (He et al., 2024) and other studies have revealed that these proteins exhibit the highest IgG levels and are considered as vaccine candidates (Sempere et al., 2021; Khan and Pichichero, 2012; Tai, 2006). This suggests that the secretion of these proteins is a common phenomenon in *S. pneumoniae* during the exponential growth phase. Notably, both HtrA and PrtA, two serine proteases in *S. pneumoniae*, were detected in our dataset. While pneumococci typically harbor up to four secreted serine proteases (HtrA, PrtA, CbpG, and SFP) depending on the strain (Ali et al., 2021a), EF3030 lacks SFP. We did not detect CbpG in our experiments including tryptic peptides with up to two cleavages, however, we found both HtrA and PrtA in the early exponential phase (OD_600_ 0.4). There was no significant fold change of HtrA during growth but PrtA showed a significant increase in the supernatant during exponential growth, further highlighting the dynamic protein secretion during this phase. The cultivation of EF3030 in the minimal medium also revealed a higher abundance of several zinc-acquisition proteins, including the AdcA and AdcAII transporters, the repressor AdcR, and the pneumococcal histidine triad (Pht) proteins. The Pht proteins, in particular, are known virulence factors in *S. pneumoniae*, with promising potential in preclinical vaccine trials aimed at preventing pneumococcal colonization and disease (Adamou et al., 2001; Godfroid et al., 2011; Denoël et al., 2011; Seiberling et al., 2012; Plumptre et al., 2013). Studies have shown that the genes encoding for AdcAII and PhtD are part of an operon regulated by the repressor AdcR. PhtD serves as an extracellular Zn^2+^ scavenger, acting as a metallophore for zinc acquisition and potentially transferring captured Zn^2+^ to the AdcAII transporter or forming a complex with AdcAII, linking zinc sensing to virulence (Loisel et al., 2011). In the presence of zinc, AdcR directly represses the *adcRCBA* genes and *adcII, phtA, phtB, phtD*, and *phtE* (Shafeeq et al., 2011).

Studies have shown that a lack of zinc (Zn) increases the risk of severe invasive pneumococcal infections and mortality. Research by (Strand et al., 2001) provides direct evidence that individuals with Zn deficiency experience more severe pneumococcal infections. Furthermore, a study by (Strand et al., 2003) reported that Zn deficiency not only worsens the infection but also weakens the body’s ability to produce specific antibodies against PspA. This leads to higher bacterial colonization in mucosal tissues, increasing the likelihood of invasive disease and death. On the other hand, research by (Bhandari et al., 2002) demonstrated that providing zinc supplements to children in developing countries significantly lowers the occurrence of pneumonia.

Given that our experiments were conducted under zinc-limiting conditions, the significant increase in Zn^2+^ transporters and Pht proteins is consistent with the bacterium’s need to scavenge zinc from its environment. This aligns with previous studies showing that Zn depletion weakens host immunity and increases pneumococcal colonization and virulence, further emphasizing the role of Zn in controlling pneumococcal infections. These findings suggest that supplementing the culture medium with zinc could reduce or eliminate the high abundance of these proteins, highlighting the adaptive mechanisms *S. pneumoniae* employs to thrive in zinc-limited conditions during infection.

Finally, a comprehensive understanding of the proteome profile during *S. pneumoniae* growth provides valuable insights into the molecular mechanisms underlying its colonization and pathogenicity. This knowledge can be leveraged to identify potential vaccine targets by highlighting key proteins involved in host interaction, immune evasion, and virulence. Additionally, it could aid in the development of strategies to block critical resistance factors, such as antibiotic efflux pumps, or surface adhesins, thereby enhancing therapeutic options. Ultimately, these findings contribute to a more detailed understanding of pneumococcal biology, which is essential for designing more effective treatments and preventive measures against this pathogen.

## 5 Conclusion

This study provides a detailed proteomic profile of *S. pneumoniae* EF3030, revealing growth-dependent changes in both the cellular and extracellular proteomes. The identified proteins, particularly those involved in transport, regulation, metabolism, and virulence, offer valuable insights into the bacterial adaptation to varying growth conditions and the potential targets for therapeutic interventions. The characterization of these proteomic shifts significantly advances our understanding of pneumococcal physiology and its response to host environments.

## Data Availability

The data presented in the study are deposited in the PRIDE repository, accession number PXD062379” (https://www.ebi.ac.uk/pride/archive/projects/PXD062379).

## References

[B1] Adamou J. E. Heinrichs J. H. Erwin A. L. Walsh W. Gayle T. Dormitzer M.. (2001). Identification and characterization of a novel family of pneumococcal proteins that are protective against sepsis. Infection Immun. 69, 949–958. doi: 10.1128/IAI.69.2.949-958.2001, PMID: 11159990 PMC97974

[B2] Ali M. Q. Kohler T. P. Burchhardt G. Wüst A. Henck N. Bolsmann R.. (2021a). Extracellular pneumococcal serine proteases affect nasopharyngeal colonization. Front. Cell. infection Microbiol. 10, 613467. doi: 10.3389/fcimb.2020.613467, PMID: 33659218 PMC7917122

[B3] Ali M. Q. Kohler T. P. Schulig L. Burchhardt G. Hammerschmidt S. (2021b). Pneumococcal extracellular serine proteases: molecular analysis and impact on colonization and disease. Front. Cell. infection Microbiol. 11, 763152. doi: 10.3389/fcimb.2021.763152, PMID: 34790590 PMC8592123

[B4] Balaban M. Bättig P. Muschiol S. Tirier S. M. Wartha F. Normark S.. (2014). Secretion of a pneumococcal type II secretion system pilus correlates with DNA uptake during transformation. Proc. Natl. Acad. Sci. United States America 111, E758–E765. doi: 10.1073/pnas.1313860111, PMID: 24550320 PMC3932930

[B5] Bättig P. Mühlemann K. (2008). Influence of the spxB gene on competence in Streptococcus pneumoniae. J. bacteriology 190, 1184–1189. doi: 10.1128/JB.01517-07, PMID: 18065543 PMC2238216

[B6] Bergmann S. Hammerschmidt S. (2006). Versatility of pneumococcal surface proteins. Microbiol. (Reading England) 152, 295–303. doi: 10.1099/mic.0.28610-0, PMID: 16436417

[B7] Bergmann S. Hammerschmidt S. (2007). Fibrinolysis and host response in bacterial infections. Thromb. Haemostasis 98, 512–520. doi: 10.1160/TH07-02-0117, PMID: 17849039

[B8] Bersch B. Bougault C. Roux L. Favier A. Vernet T. Durmort C. (2013). New insights into histidine triad proteins: solution structure of a Streptococcus pneumoniae PhtD domain and zinc transfer to AdcAII. PloS One 8 (11), e81168. doi: 10.1371/journal.pone.0081168, PMID: 24312273 PMC3842936

[B9] Bhandari N. Bahl R. Taneja S. Strand T. Mølbak K. Johan Ulvik R.. (2002). Substantial reduction in severe diarrheal morbidity by daily zinc supplementation in young north Indian children. Pediatrics 109 (6), pp.e86-e86. doi: 10.1136/bmj.324.7350.1358, PMID: 12042580

[B10] Bogaert D. de Groot R. Hermans P. W. M. (2004). Streptococcus pneumoniae colonisation: the key to pneumococcal disease. Lancet Infect. Dis. 4, 144–154. doi: 10.1016/S1473-3099(04)00938-7, PMID: 14998500

[B11] Brown J. S. Gilliland S. M. Holden D. W. (2001). A Streptococcus pneumoniae pathogenicity island encoding an ABC transporter involved in iron uptake and virulence. Mol. Microbiol. 40, 572–585. doi: 10.1046/j.1365-2958.2001.02414.x, PMID: 11359564

[B12] Brown J. S. Gilliland S. M. Ruiz-Albert J. Holden D. W. (2002). Characterization of pit, a Streptococcus pneumoniae iron uptake ABC transporter. Infection Immun. 70, 4389–4398. doi: 10.1128/IAI.70.8.4389-4398.2002, PMID: 12117949 PMC128127

[B13] Burghout P. Zomer A. van der Gaast-de Jongh C. E. Janssen-Megens E. M. Françoijs K.-J. Stunnenberg H. G.. (2013). Streptococcus pneumoniae folate biosynthesis responds to environmental CO2 levels. J. bacteriology 195, 1573–1582. doi: 10.1128/JB.01942-12, PMID: 23354753 PMC3624543

[B14] Cartwright K. (2002). Pneumococcal disease in western Europe: burden of disease, antibiotic resistance and management. Eur. J. Pediatr. 161, 188–195. doi: 10.1007/s00431-001-0907-3, PMID: 12014384

[B15] Carvalho S. M. Kloosterman T. G. Kuipers O. P. Neves A. R. (2011). CcpA ensures optimal metabolic fitness of Streptococcus pneumoniae. PloS One 6, e26707. doi: 10.1371/journal.pone.0026707, PMID: 22039538 PMC3198803

[B16] Carvalho S. M. Kuipers O. P. Neves A. R. (2013). Environmental and nutritional factors that affect growth and metabolism of the pneumococcal serotype 2 strain D39 and its nonencapsulated derivative strain R6. PloS One 8, e58492. doi: 10.1371/journal.pone.0058492, PMID: 23505518 PMC3591343

[B17] Chao Y. Marks L. R. Pettigrew M. M. Hakansson A. P. (2014). Streptococcus pneumoniae biofilm formation and dispersion during colonization and disease. Front. Cell. infection Microbiol. 4, 194. doi: 10.3389/fcimb.2014.00194, PMID: 25629011 PMC4292784

[B18] Claverys J.-P. Prudhomme M. Martin B. (2006). Induction of competence regulons as a general response to stress in gram-positive bacteria. Annu. Rev. Microbiol. 60, 451–475. doi: 10.1146/annurev.micro.60.080805.142139, PMID: 16771651

[B19] De S. Hakansson A. P. (2023). Measuring niche-associated metabolic activity in planktonic and biofilm bacteria. Methods and Protocols (pp. 3-32). New York, NY: Springer US. doi: 10.1007/978-1-0716-3243-7_1, PMID: 37258957

[B20] Denoël P. Philipp M. T. Doyle L. Martin D. Carletti G. Poolman J. T. (2011). A proteinbased pneumococcal vaccine protects rhesus macaques from pneumonia after experimental infection with Streptococcus pneumoniae. Vaccine 29, 5495–5501. doi: 10.1016/j.vaccine.2011.05.051, PMID: 21624422 PMC5061031

[B21] Deutsch E. W. Bandeira N. Perez-Riverol Y. Sharma V. Carver J. J. Mendoza L.. (2023). The ProteomeXchange consortium at 10 years: 2023 update. Nucleic Acids Res. 51, D1539–D1548. doi: 10.1093/nar/gkac1040, PMID: 36370099 PMC9825490

[B22] Eijkelkamp B. A. Morey J. R. Ween M. P. Ong C. Y. McEwan A. G. Paton J. C.. (2014). Extracellular zinc competitively inhibits manganese uptake and compromises oxidative stress management in Streptococcus pneumoniae. PloS One 9, e89427. doi: 10.1371/journal.pone.0089427, PMID: 24558498 PMC3928430

[B23] Eldholm V. Johnsborg O. Haugen K. Ohnstad H. S. Håvarstein L. S. (2009). Fratricide in Streptococcus pneumoniae: contributions and role of the cell wall hydrolases CbpD, LytA and LytC. Microbiol. (Reading England) 155, 2223–2234. doi: 10.1099/mic.0.026328-0, PMID: 19389766

[B24] Elm C. Rohde M. Vaerman J. P. Chhatwal G. S. Hammerschmidt S. (2004). Characterization of the interaction of the pneumococcal surface protein SpsA with the human polymeric immunoglobulin receptor (hpIgR). Indian Journal of Medical Research, 119, pp.61-65., PMID: 15232164

[B25] Fatykhova D. Fritsch V. N. Siebert K. Methling K. Lalk M. Busche T.. (2024). Microenvironmental acidification by pneumococcal sugar consumption fosters barrier disruption and immune suppression in the human alveolus. Eur. Respir. J. 64. doi: 10.1183/13993003.01983-2023, PMID: 39231629 PMC11635383

[B26] Frankel M. B. Hendrickx A. P. A. Missiakas D. M. Schneewind O. (2011). LytN, a murein hydrolase in the cross-wall compartment of Staphylococcus aureus, is involved in proper bacterial growth and envelope assembly. J. Biol. Chem. 286, 32593–32605. doi: 10.1074/jbc.M111.258863, PMID: 21784864 PMC3173183

[B27] Fritsch K. J. Krüger L. Handtke S. Kohler T. P. Ozhiganova A. Jahn K.. (2024). Pneumococcal neuraminidases increase platelet killing by pneumolysin. Thromb. haemostasis 125, 243–254. doi: 10.1055/a-2369-8680, PMID: 39029905

[B28] Gilley R. P. Orihuela C. J. (2014). Pneumococci in biofilms are non-invasive: implications on nasopharyngeal colonization. Front. Cell. infection Microbiol. 4, 163. doi: 10.3389/fcimb.2014.00163, PMID: 25414838 PMC4222220

[B29] Godfroid F. Hermand P. Verlant V. Denoël P. Poolman J. T. (2011). Preclinical evaluation of the Pht proteins as potential cross-protective pneumococcal vaccine antigens. Infection Immun. 79, 238–245. doi: 10.1128/IAI.00378-10, PMID: 20956575 PMC3019885

[B30] Guiral S. Mitchell T. J. Martin B. Claverys J. (2005). Competence-programmed predation of noncompetent cells in the human pathogen Streptococcus pneumoniae: genetic requirements. Proceedings of the National Academy of Sciences, 102 (24), p.68. doi: 10.1073/pnas.0500879102, PMID: 15928084 PMC1150823

[B31] Gupta R. Shah P. Swiatlo E. (2009). Differential gene expression in Streptococcus pneumoniae in response to various iron sources. Microbial pathogenesis 47, 101–109. doi: 10.1016/j.micpath.2009.05.003, PMID: 19464356

[B32] Haft D. H. Selengut J. D. Richter R. A. Harkins D. Basu M. K. Beck E. (2013). TIGRFAMs and genome properties in 2013. Nucleic Acids Res. 41, D387–D395. doi: 10.1093/nar/gks1234, PMID: 23197656 PMC3531188

[B33] Hall-Stoodley L. Costerton J. W. Stoodley P. (2004). Bacterial biofilms: from the natural environment to infectious diseases. Nat. Rev. Microbiol. 2, 95–108. doi: 10.1038/nrmicro821, PMID: 15040259

[B34] Hammerschmidt S. (2006). Adherence molecules of pathogenic pneumococci. Curr. Opin. Microbiol. 9, 12–20. doi: 10.1016/j.mib.2005.11.001, PMID: 16338163

[B35] Härtel T. Eylert E. Schulz C. Petruschka L. Gierok P. Grubmüller S.. (2012). Characterization of central carbon metabolism of Streptococcus pneumoniae by isotopologue profiling. J. Biol. Chem. 287, 4260–4274. doi: 10.1074/jbc.M111.304311, PMID: 22167202 PMC3281726

[B36] Härtel T. Klein M. Koedel U. Rohde M. Petruschka L. Hammerschmidt S. (2011). Impact of glutamine transporters on pneumococcal fitness under infection-related conditions. Infection Immun. 79, 44–58. doi: 10.1128/IAI.00855-10, PMID: 21078855 PMC3019899

[B37] He S. W. J. Voß F. Nicolaie M. A. Brummelman J. van de Garde M. D. B. Bijvank E.. (2024). Serological profiling of pneumococcal proteins reveals unique patterns of acquisition, maintenance, and waning of antibodies throughout life. J. Infect. Dis. 230, e1299–e1310. doi: 10.1093/infdis/jiae216, PMID: 38679601 PMC11646596

[B38] Hendriksen W. T. Bootsma H. J. Estevão S. Hoogenboezem T. de Jong A. de Groot R.. (2007). CodY of Streptococcus pneumoniae: link between nutritional gene regulation and colonization. J. bacteriology 190, 590–601. doi: 10.1128/JB.00917-07, PMID: 18024519 PMC2223708

[B39] Im H. Kruckow K. L. D'Mello A. Ganaie F. Martinez E. Luck J. N.. (2022). Anatomical Site-Specific Carbohydrate Availability Impacts Streptococcus pneumoniae Virulence and Fitness during Colonization and Disease. Infection Immun. 90, e0045121. doi: 10.1128/IAI.00451-21, PMID: 34748366 PMC8788743

[B40] Iyer R. Baliga N. S. Camilli A. (2005). Catabolite control protein A (CcpA) contributes to virulence and regulation of sugar metabolism in Streptococcus pneumoniae. J. bacteriology 187, 8340–8349. doi: 10.1128/JB.187.24.8340-8349.2005, PMID: 16321938 PMC1317011

[B41] Jacoby W. G. (2000). Loess: a nonparametric, graphical tool for depicting relationships between variables. Electoral studies, 19 (4), pp. 577-613. doi: 10.1016/S0261-3794(99)00028-1

[B42] Jensch I. Gámez G. Rothe M. Ebert S. Fulde M. Somplatzki D.. (2010). PavB is a surface-exposed adhesin of Streptococcus pneumoniae contributing to nasopharyngeal colonization and airways infections. Mol. Microbiol. 77, 22–43. doi: 10.1111/j.1365-2958.2010.07189.x, PMID: 20444103

[B43] Johnston C. Bootsma H. J. Aldridge C. Manuse S. Gisch N. Schwudke D.. (2015). Co-inactivation of GlnR and CodY regulators impacts pneumococcal cell wall physiology. PloS One 10, e0123702. doi: 10.1371/journal.pone.0123702, PMID: 25901369 PMC4406557

[B44] Junges R. Maienschein-Cline M. Morrison D. A. Petersen F. C. (2019). Complete genome sequence of streptococcus pneumoniae serotype 19F strain EF3030. Microbiol. resource announcements 8 (19), pp.10-1128. doi: 10.1128/MRA.00198-19, PMID: 31072896 PMC6509521

[B45] Kallio A. Sepponen K. Hermand P. Denoël P. Godfroid F. Melin M. (2014). Role of Pht proteins in attachment of Streptococcus pneumoniae to respiratory epithelial cells. Infection Immun. 82, 1683–1691. doi: 10.1128/IAI.00699-13, PMID: 24491577 PMC3993382

[B46] Kanwal S. Jensch I. Palm G. J. Brönstrup M. Rohde M. Kohler T. P.. (2017). Mapping the recognition domains of pneumococcal fibronectin-binding proteins PavA and PavB demonstrates a common pattern of molecular interactions with fibronectin type III repeats. Mol. Microbiol. 105, 839–859. doi: 10.1111/mmi.2017.105.issue-6, PMID: 28657670

[B47] Kausmally L. Johnsborg O. Lunde M. Knutsen E. Håvarstein L. S. (2005). Cholinebinding protein D (CbpD) in Streptococcus pneumoniae is essential for competence-induced cell lysis. J. bacteriology 187, 4338–4345. doi: 10.1128/JB.187.13.4338-4345.2005, PMID: 15968042 PMC1151764

[B48] Khan M. N. Pichichero M. E. (2012). Vaccine candidates PhtD and PhtE of Streptococcus pneumoniae are adhesins that elicit functional antibodies in humans. Vaccine 30, 2900–2907. doi: 10.1016/j.vaccine.2012.02.023, PMID: 22349524 PMC3490617

[B49] King S. J. (2010). Pneumococcal modification of host sugars: a major contributor to colonization of the human airway. Mol. Oral. Microbiol. 25, 15–24. doi: 10.1111/j.2041-1014.2009.00564.x, PMID: 20331791

[B50] Kohler S. Voß F. Gómez Mejia A. Brown J. S. Hammerschmidt S. (2016). Pneumococcal lipoproteins involved in bacterial fitness, virulence, and immune evasion. FEBS Lett. 590, 3820–3839. doi: 10.1002/feb2.2016.590.issue-21, PMID: 27508940

[B51] Korotkevich G. Sukhov V. Budin N. Shpak B. Artyomov M. N. Sergushichev A. (2016). Fast gene set enrichment analysis. biorxiv, p.060012. doi: 10.1101/060012

[B52] Krismer B. Liebeke M. Janek D. Nega M. Rautenberg M. Hornig G.. (2014). Nutrient limitation governs Staphylococcus aureus metabolism and niche adaptation in the human nose. PloS Pathog. 10, e1003862. doi: 10.1371/journal.ppat.1003862, PMID: 24453967 PMC3894218

[B53] Lane J. R. Tata M. Briles D. E. Orihuela C. J. (2022). A jack of all trades: the role of pneumococcal surface protein A in the pathogenesis of streptococcus pneumoniae. Front. Cell. infection Microbiol. 12, 826264. doi: 10.3389/fcimb.2022.826264, PMID: 35186799 PMC8847780

[B54] Lanie J. A. Ng W.-L. Kazmierczak K. M. Andrzejewski T. M. Davidsen T. M. Wayne K. J.. (2007). Genome sequence of Avery's virulent serotype 2 strain D39 of Streptococcus pneumoniae and comparison with that of unencapsulated laboratory strain R6. J. bacteriology 189, 38–51. doi: 10.1128/JB.01148-06, PMID: 17041037 PMC1797212

[B55] Lee K.-J. Bae S.-M. Lee M.-R. Yeon S.-M. Lee Y.-H. Kim K.-S. (2006). Proteomic analysis of growth phase-dependent proteins of Streptococcus pneumoniae. Proteomics 6, 1274–1282. doi: 10.1002/pmic.200500415, PMID: 16429463

[B56] Leonard A. Gierok P. Methling K. Gómez-Mejia A. Hammerschmidt S. Lalk M. (2018). Metabolic inventory of Streptococcus pneumoniae growing in a chemical defined environment. Int. J. Med. Microbiol. IJMM 308, 705–712. doi: 10.1016/j.ijmm.2018.01.001, PMID: 29398251

[B57] Liu X. Li J.-W. Feng Z. Luo Y. Veening J.-W. Zhang J.-R. (2017). Transcriptional repressor PtvR regulates phenotypic tolerance to vancomycin in streptococcus pneumoniae. J. bacteriology 199 (14), pp.10-1128. doi: 10.1128/JB.00054-17, PMID: 28484041 PMC5494751

[B58] Liu Y. Zeng Y. Huang Y. Gu L. Wang S. Li C.. (2019). HtrA-mediated selective degradation of DNA uptake apparatus accelerates termination of pneumococcal transformation. Mol. Microbiol. 112, 1308–1325. doi: 10.1111/mmi.v112.4, PMID: 31396996

[B59] Loisel E. Chimalapati S. Bougault C. Imberty A. Gallet B. Di Guilmi A. M.. (2011). Biochemical characterization of the histidine triad protein PhtD as a cell surface zinc-binding protein of pneumococcus. Biochemistry 50, 3551–3558. doi: 10.1021/bi200012f, PMID: 21425866

[B60] Marquart M. E. (2021). Pathogenicity and virulence of Streptococcus pneumoniae: Cutting to the chase on proteases. Virulence 12, 766–787. doi: 10.1080/21505594.2021.1889812, PMID: 33660565 PMC7939560

[B61] Martin J. E. Lisher J. P. Winkler M. E. Giedroc D. P. (2017). Perturbation of manganese metabolism disrupts cell division in Streptococcus pneumoniae. Mol. Microbiol. 104, 334–348. doi: 10.1111/mmi.2017.104.issue-2, PMID: 28127804 PMC5380469

[B62] Michalik S. Hammer E. Steil L. Salazar M. G. Hentschker C. Surmann K.. (2025). SpectroPipeR-a streamlining post Spectronaut® DIA-MS data analysis R package. Bioinf. (Oxford England) 41 (3), btaf086. doi: 10.1093/bioinformatics/btaf086, PMID: 39985446 PMC11893148

[B63] Moreno J. Nielsen H. Winther O. Teufel F. (2024). Predicting the subcellular location of prokaryotic proteins with DeepLocPro. Bioinformatics, 40 (12), p.btae677. doi: 10.1101/2024.01.04.574157, PMID: 39540738 PMC11645106

[B64] Morsczeck C. Prokhorova T. Sigh J. Pfeiffer M. Bille-Nielsen M. Petersen J.. (2008). Streptococcus pneumoniae: proteomics of surface proteins for vaccine development. Clin. Microbiol. infection 14, 74–81. doi: 10.1111/j.1469-0691.2007.01878.x, PMID: 18034862

[B65] Narciso A. R. Dookie R. Nannapaneni P. Normark S. Henriques-Normark B. (2024). Streptococcus pneumoniae epidemiology, pathogenesis and control. Nat. Rev. Microbiol. 23 (4), pp.256-271. doi: 10.1038/s41579-024-01116-z, PMID: 39506137

[B66] Nelson A. L. Roche A. M. Gould J. M. Chim K. Ratner A. J. Weiser J. N. (2007). Capsule enhances pneumococcal colonization by limiting mucus-mediated clearance. Infection Immun. 75, 83–90. doi: 10.1128/IAI.01475-06, PMID: 17088346 PMC1828419

[B67] Novichkov P. S. Kazakov A. E. Ravcheev D. A. Leyn S. A. Kovaleva G. Y. Sutormin R. A.. (2013). A resource for genome-scale exploration of transcriptional regulation in bacteria. BMC genomics, 14, pp.1-12. doi: 10.1186/1471-2164-14-745, PMID: 24175918 PMC3840689

[B68] Ong C. Y. Potter A. J. Trappetti C. Walker M. J. Jennings M. P. Paton J. C.. (2013). Interplay between manganese and iron in pneumococcal pathogenesis: role of the orphan response regulator RitR. Infection Immun. 81, 421–429. doi: 10.1128/IAI.00805-12, PMID: 23184523 PMC3553810

[B69] Paulikat A. D. Schwudke D. Hammerschmidt S. Voß F. (2024). Lipidation of pneumococcal proteins enables activation of human antigen-presenting cells and initiation of an adaptive immune response. Front. Immunol. 15, 1392316. doi: 10.3389/fimmu.2024.1392316, PMID: 38711516 PMC11070533

[B70] Pérez-Dorado I. Galan-Bartual S. Hermoso J. A. (2012). Pneumococcal surface proteins: when the whole is greater than the sum of its parts. Mol. Oral. Microbiol. 27, 221–245. doi: 10.1111/j.2041-1014.2012.00655.x, PMID: 22759309

[B71] Perez-Riverol Y. Bandla C. Kundu D. J. KamatChinathan S. Bai J. Hewapathirana S.. (2024). The PRIDE database at 20 years: 2025 update. Nucleic Acids Res. 53, D543–D553., PMID: 39494541 10.1093/nar/gkae1011 PMC11701690

[B72] Perez-Riverol Y. Xu Q.-W. Wang R. Uszkoreit J. Griss J. Sanchez A.. (2016). PRIDE inspector toolsuite: moving toward a universal visualization tool for proteomics data standard formats and quality assessment of proteomeXchange datasets. Mol. Cell. Proteomics MCP 15, 305–317. doi: 10.1074/mcp.O115.050229, PMID: 26545397 PMC4762524

[B73] Pericone C. D. Overweg K. Hermans P. W. Weiser J. N. (2000). Inhibitory and bactericidal effects of hydrogen peroxide production by Streptococcus pneumoniae on other inhabitants of the upper respiratory tract. Infection Immun. 68, 3990–3997. doi: 10.1128/IAI.68.7.3990-3997.2000, PMID: 10858213 PMC101678

[B74] Pimentel Z. T. Zhang Y. (2018). Evolution of the natural transformation protein, comEC, in bacteria. Front. Microbiol. 9, 2980. doi: 10.3389/fmicb.2018.02980, PMID: 30627116 PMC6299819

[B75] Plumptre C. D. Ogunniyi A. D. Paton J. C. (2013). Surface association of Pht proteins of Streptococcus pneumoniae. Infection Immun. 81, 3644–3651. doi: 10.1128/IAI.00562-13, PMID: 23876799 PMC3811752

[B76] Reder A. Hentschker C. Steil L. Gesell Salazar M. Hammer E. Dhople V. M.. (2024). MassSpecPreppy-An end-to-end solution for automated protein concentration determination and flexible sample digestion for proteomics applications. Proteomics 24, e2300294. doi: 10.1002/pmic.202300294, PMID: 37772677

[B77] Rigden D. J. Galperin M. Y. Jedrzejas M. J. (2003). Analysis of structure and function of putative surface-exposed proteins encoded in the Streptococcus pneumoniae genome: a bioinformatics-based approach to vaccine and drug design. Crit. Rev. Biochem. Mol. Biol. 38, 143–168. doi: 10.1080/713609215, PMID: 12749697

[B78] Sanchez-Rosario Y. Johnson M. D. L. (2021). Media matters, examining historical and modern streptococcus pneumoniae growth media and the experiments they affect. Front. Cell. infection Microbiol. 11, 613623. doi: 10.3389/fcimb.2021.613623, PMID: 33834003 PMC8021847

[B79] Schulz C. Gierok P. Petruschka L. Lalk M. Mäder U. Hammerschmidt S. (2014). Regulation of the arginine deiminase system by ArgR2 interferes with arginine metabolism and fitness of Streptococcus pneumoniae. mBio 5 (6), pp.10-1128. doi: 10.1128/mBio.01858-14, PMID: 25538192 PMC4278536

[B80] Schulz C. Hammerschmidt S. (2013). Exploitation of physiology and metabolomics to identify pneumococcal vaccine candidates. Expert review of vaccines, 12 (9), pp.1061-1075. doi: 10.1586/14760584.2013.824708, PMID: 24053399

[B81] Schwanhäusser B. Busse D. Li N. Dittmar G. Schuchhardt J. Wolf J.. (2011). Global quantification of mammalian gene expression control. Nature 473, 337–342. doi: 10.1038/nature10098, PMID: 21593866

[B82] Seiberling M. Bologa M. Brookes R. Ochs M. Go K. Neveu D.. (2012). Safety and immunogenicity of a pneumococcal histidine triad protein D vaccine candidate in adults. Vaccine 30, 7455–7460. doi: 10.1016/j.vaccine.2012.10.080, PMID: 23131206

[B83] Sempere J. Llamosí M. Del Río Menéndez I. López Ruiz B. Domenech M. González-Camacho F. (2021). Pneumococcal choline-binding proteins involved in virulence as vaccine candidates. Vaccines 9 (2), p.181. doi: 10.3390/vaccines9020181, PMID: 33672701 PMC7924319

[B84] Shafeeq S. Kloosterman T. G. Kuipers O. P. (2011). Transcriptional response of Streptococcus pneumoniae to Zn2+) limitation and the repressor/activator function of AdcR. Metallomics integrated biometal Sci. 3, 609–618. doi: 10.1039/c1mt00030f, PMID: 21603707

[B85] Simell B. Auranen K. Käyhty H. Goldblatt D. Dagan R. O'Brien K. L. (2012). The fundamental link between pneumococcal carriage and disease. Expert Rev. Vaccines 11, 841–855. doi: 10.1586/erv.12.53, PMID: 22913260

[B86] Strand T. A. Briles D. E. Gjessing H. K. Maage A. Bhan M. K. Sommerfelt H. (2001). Pneumococcal pulmonary infection, septicaemia and survival in young zinc-depleted mice. Br. J. Nutr. 86, 301–306. doi: 10.1079/BJN2001399, PMID: 11502245

[B87] Strand T. A. Hollingshead S. K. Julshamn K. Briles D. E. Blomberg B. Sommerfelt H. (2003). Effects of zinc deficiency and pneumococcal surface protein a immunization on zinc status and the risk of severe infection in mice. Infection Immun. 71, 2009–2013. doi: 10.1128/IAI.71.4.2009-2013.2003, PMID: 12654820 PMC152078

[B88] Subramanian A. Tamayo P. Mootha V. K. Mukherjee S. Ebert B. L. Gillette M. A.. (2005). Gene set enrichment analysis: A knowledge-based approach for interpreting genome-wide expression profiles. Proceedings of the National Academy of Sciences, 102 (43), pp.15545-15550. doi: 10.1073/pnas.0506580102, PMID: 16199517 PMC1239896

[B89] Suomi T. Elo L. L. (2017). Enhanced differential expression statistics for data-independent acquisition proteomics. Sci. Rep. 7, 5869. doi: 10.1038/s41598-017-05949-y, PMID: 28724900 PMC5517573

[B90] Tai S. S. (2006). Streptococcus pneumoniae protein vaccine candidates: properties, activities and animal studies. Crit. Rev. Microbiol. 32, 139–153. doi: 10.1080/10408410600822942, PMID: 16893751

[B91] Terrasse R. Amoroso A. Vernet T. Di Guilmi A. M. (2015). Streptococcus pneumoniae GAPDH is released by cell lysis and interacts with peptidoglycan. PloS One 10, e0125377. doi: 10.1371/journal.pone.0125377, PMID: 25927608 PMC4415926

[B92] van Beek L. F. Surmann K. van Berg Saparoea H. B. Houben D. Jong W. S. P. Hentschker C.. (2020). Exploring metal availability in the natural niche of Streptococcus pneumoniae to discover potential vaccine antigens. Virulence 11, 1310–1328. doi: 10.1080/21505594.2020.1825908, PMID: 33017224 PMC7550026

[B93] Voß F. Kohler T. P. Meyer T. Abdullah M. R. van Opzeeland F. J. Saleh M.. (2018). Intranasal vaccination with lipoproteins confers protection against pneumococcal colonisation. Front. Immunol. 9. doi: 10.3389/fimmu.2018.02405, PMID: 30405609 PMC6202950

[B94] Weiser J. N. Ferreira D. M. Paton J. C. (2018). Streptococcus pneumoniae: transmission, colonization and invasion. Nat. Rev. Microbiol. 16, 355–367. doi: 10.1038/s41579-018-0001-8, PMID: 29599457 PMC5949087

[B95] Weiser J. N. Kapoor M. (1999). Effect of intrastrain variation in the amount of capsular polysaccharide on genetic transformation of Streptococcus pneumoniae: implications for virulence studies of encapsulated strains. Infection Immun. 67, 3690–3692. doi: 10.1128/IAI.67.7.3690-3692.1999, PMID: 10377162 PMC116567

[B96] Weyder M. Prudhomme M. Bergé M. Polard P. Fichant G. (2018). Dynamic modeling of Streptococcus pneumoniae competence provides regulatory mechanistic insights into its tight temporal regulation. Front. Microbiol. 9, 1637. doi: 10.3389/fmicb.2018.01637, PMID: 30087661 PMC6066662

[B97] Winkler M. E. Morrison D. A. (2019). Competence beyond genes: filling in the details of the pneumococcal competence transcriptome by a systems approach. J. hygiene 27, 113–159. doi: 10.1128/JB.00238-19, PMID: 30988030 PMC6560134

[B98] Yao K.-H. Yang Y.-H. (2008). Streptococcus pneumoniae diseases in Chinese children: past, present and future. Vaccine 26, 4425–4433. doi: 10.1016/j.vaccine.2008.06.052, PMID: 18602435

[B99] Zhang Y. Zhang J. Xiao J. Wang H. Yang R. Guo X.. (2023). comCDE (Competence) operon is regulated by CcpA in Streptococcus pneumoniae D39. Microbiol. Spectr. 11, e0001223. doi: 10.1128/spectrum.00012-23, PMID: 37036382 PMC10269683

