# Supplementary material for: The global proteome of Streptococcus pneumoniae EF3030 under nutrient-defined in vitro conditions
Source: Front Cell Infect Microbiol. 2025 Jul 11;15:1606161. doi: 10.3389/fcimb.2025.1606161 (PMC12289677; doi:10.3389/fcimb.2025.1606161)
Supplement: Supplementary file 1 [file DataSheet1.pdf]

Supplementary Material

The global proteome of *Streptococcus pneumoniae* EF3030 under nutrient-defined *in vitro* conditions

Supradipta De[1], Larissa M. Busch[2], Gerhard Burchhardt[1], Manuela Gesell Salazar[2], Rabea Schlüter[3], Leif Steil[2], Uwe Völker[2], and Sven Hammerschmidt[1]*

***Correspondence:** Sven Hammerschmidt, sven.hammerschmidt@uni-greifswald.de

1 Supplementary Figures

A

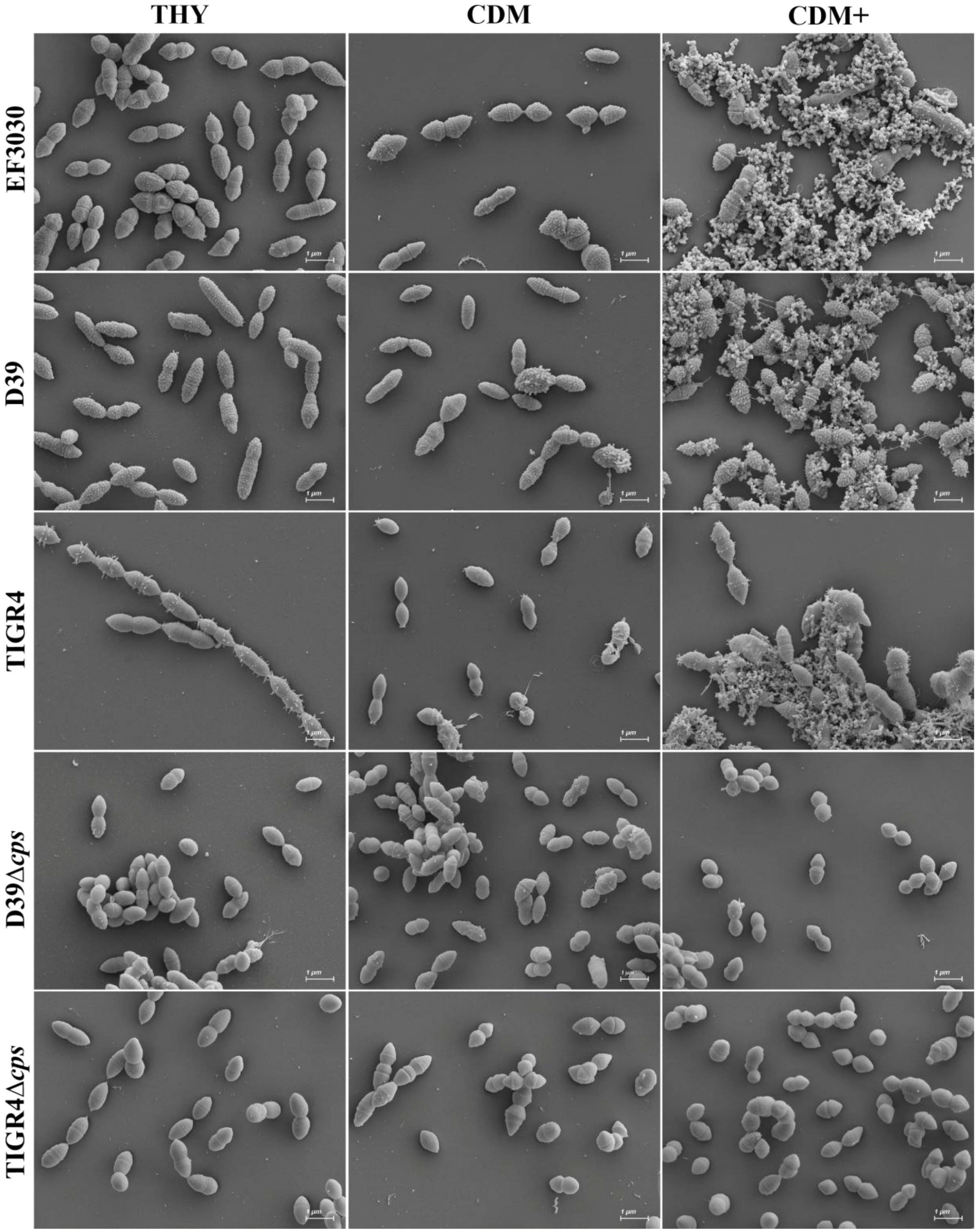

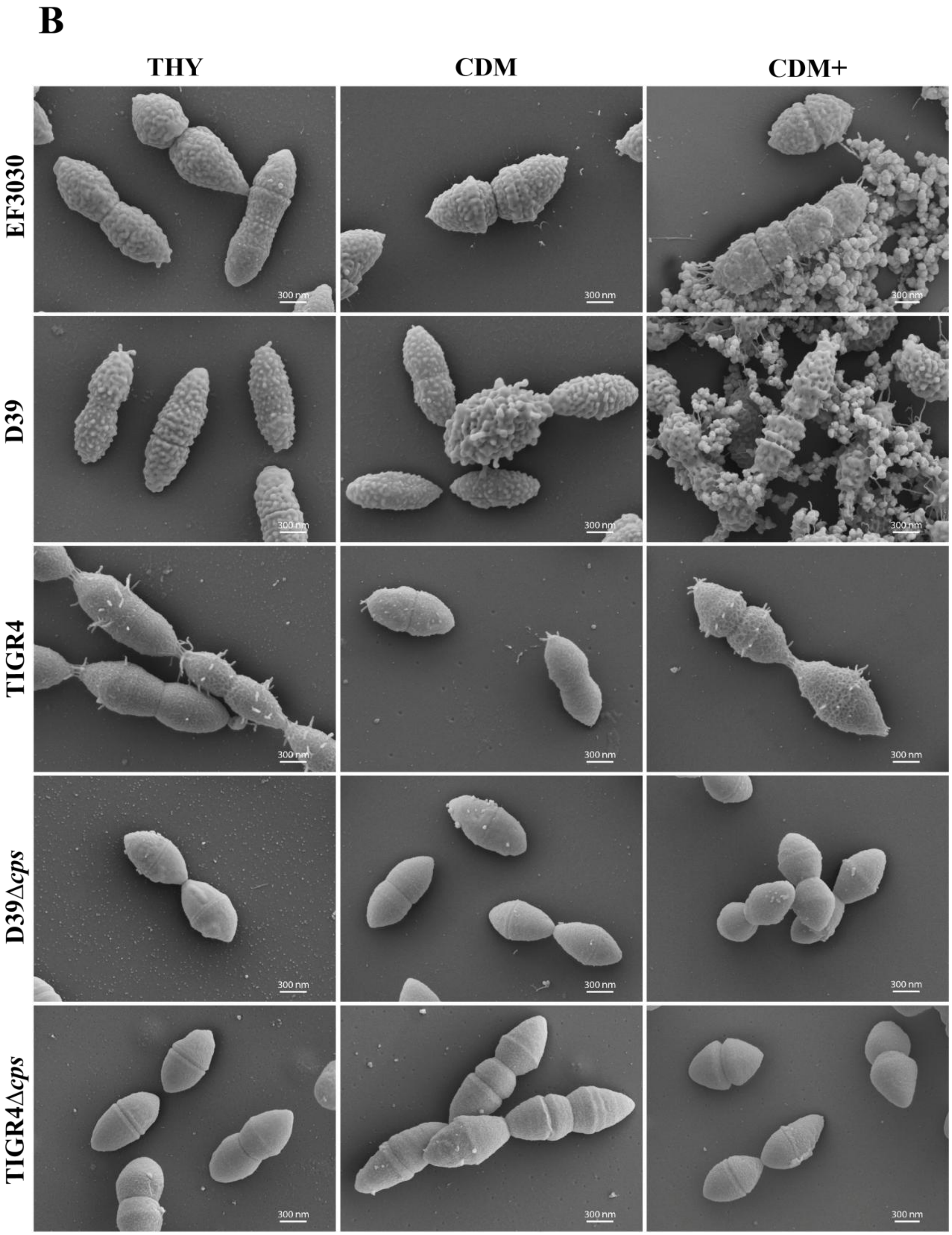

Supplementary Figure S1: Scanning electron micrographs of *S. pneumoniae* cultivated in THY, CDM, or CDM**+.** The micrographs were taken at a magnification of 10,000x, scale bars = 1 µm (A) and at a higher magnification of 30,000x, scales bar = 300 nm (B).

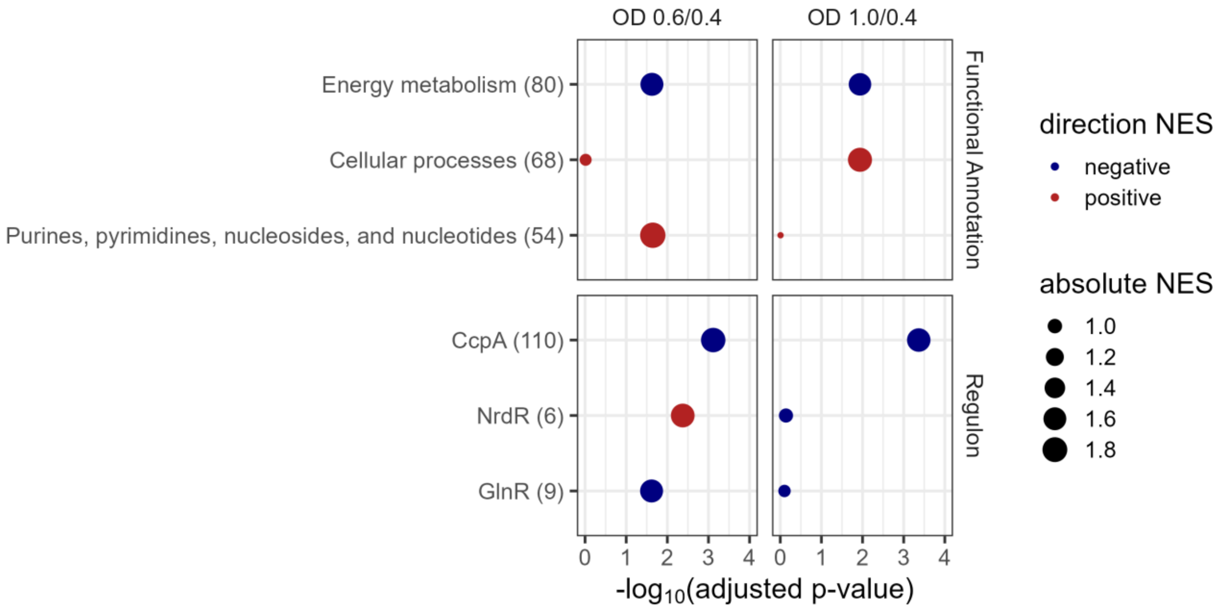

Supplementary Figure S2: Gene set enrichment analyses (GSEA) comparing different biological functions and regulatory networks between conditions. X-axis represents $\log_{10}$ (adjusted p-value), y-axis shows Functional annotation (top panel) and Regulon (bottom panel). Each dot represents a pathway or regulon indicated by “Red: positively enriched” and “Blue: negatively enriched”. The size of the dots reflects the absolute Normalized Enrichment Score (NES) indicating magnitude of enrichment.

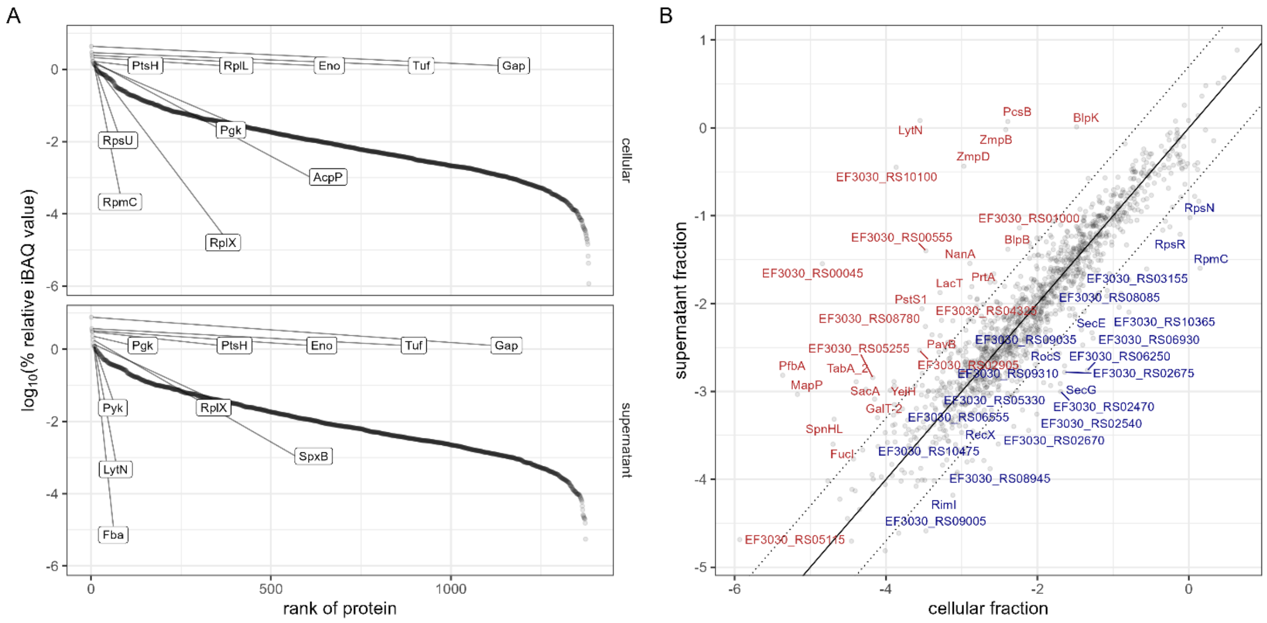

Supplementary Figure S3: Protein composition of the cellular and supernatant fraction of the proteome at OD_{600} 1.0. (A) Display of the range of detected abundances scaled as relative iBAQ values in the cellular and supernatant fraction. The top 10 most abundant proteins are labelled. (B) Comparison of the log_{10}-scaled relative iBAQ value per protein in the cellular and supernatant fraction. Solid line displays perfect correlation; dotted lines display 5-fold difference of relative abundance between both fractions. Proteins differing at least 10-fold are labelled. Proteins in higher relative abundance in the supernatant are coloured red and proteins in higher relative abundance in the cellular fraction are coloured blue.

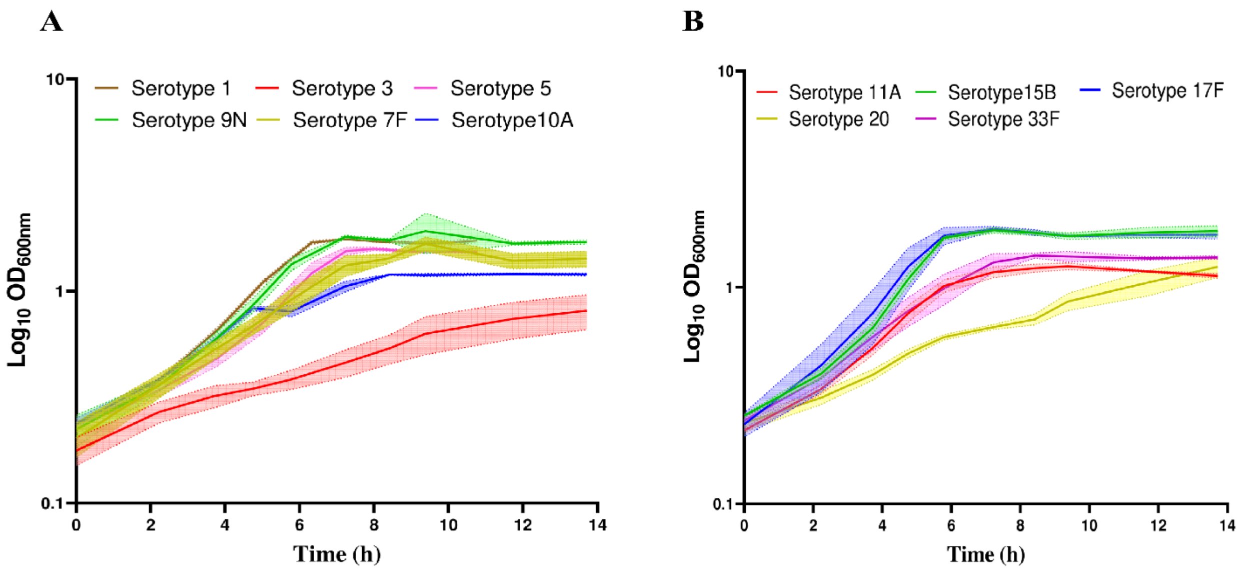

Supplementary Figure S4: Growth kinetics of *Streptococcus pneumoniae* invasive clinical strains representing different serotypes in CDM+ medium. Representative strains were cultured in chemically defined medium with supplementation (CDM+), and growth was monitored measuring optical density at 600 nm (OD_{600}). Values are plotted as Log_{10} OD_{600} against time (hours). Data represent the mean ± standard deviation of three biological replicates. Distinct growth profiles among serotypes were observed.

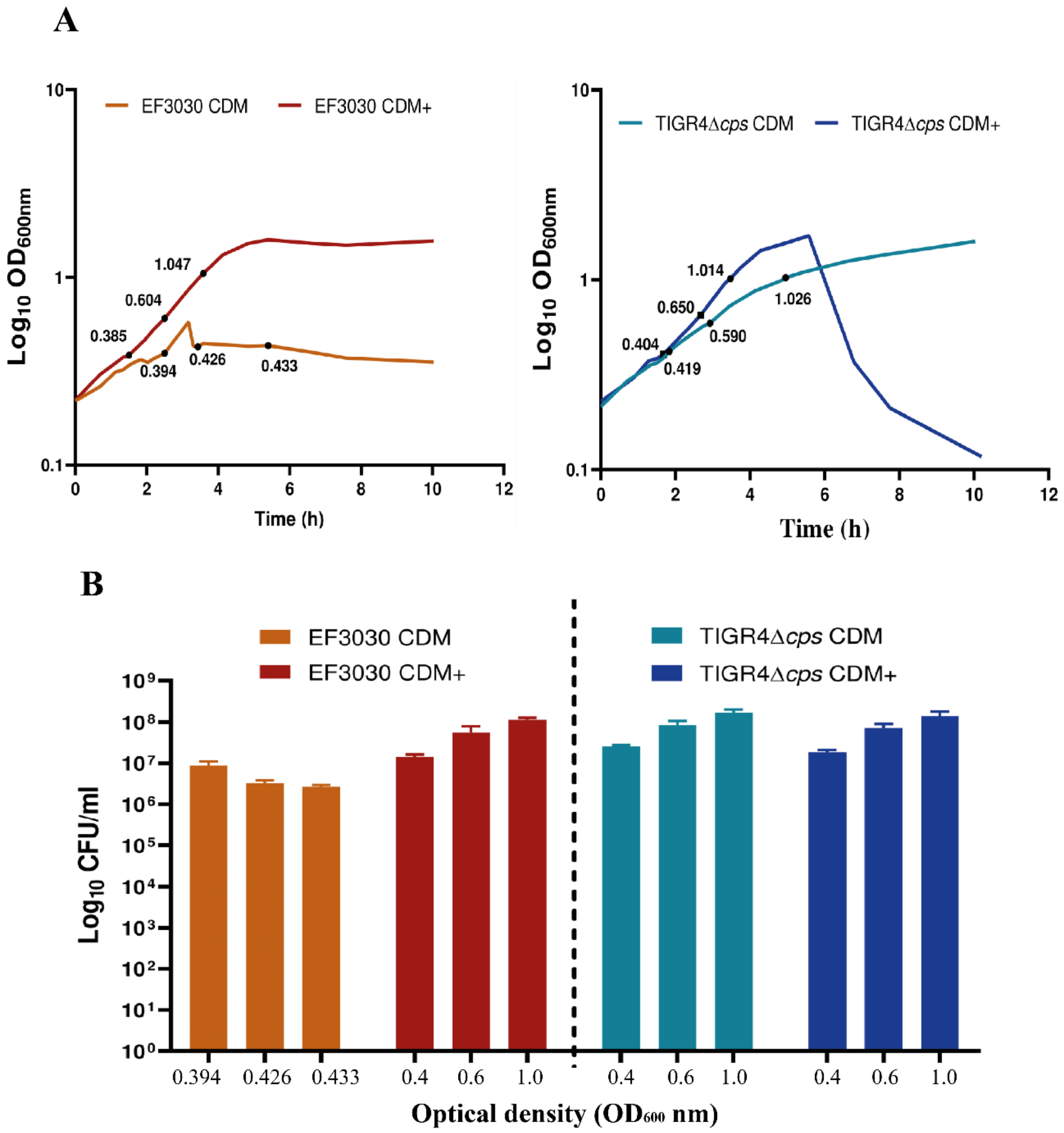

Supplementary Figure S5: Growth dynamics and viability of *Streptococcus pneumoniae* EF3030 and TIGR4Δ*cps* in chemically defined media. (A) Growth curves of *S. pneumoniae* EF3030 and TIGR4Δ*cps* cultured in CDM or modified CDM+. The optical density (OD_{600}) was monitored over time. OD values at specific time points indicate when samples were harvested for viable cell enumeration. (B) Colony-forming units (CFU/mL) determined at selected growth phases corresponding to panel A. Data are presented as mean ± standard deviation from biological triplicates. Samples were collected during early ($OD_{600} \approx 0.4$), mid ($OD_{600} \approx 0.6$), and late ($OD_{600} \approx 1.0$) exponential growth phases. However, strain EF3030 did not reach an OD_{600} of 1.0; therefore, samples for this strain were collected at random time points to assess colony-forming unit (CFU) counts.

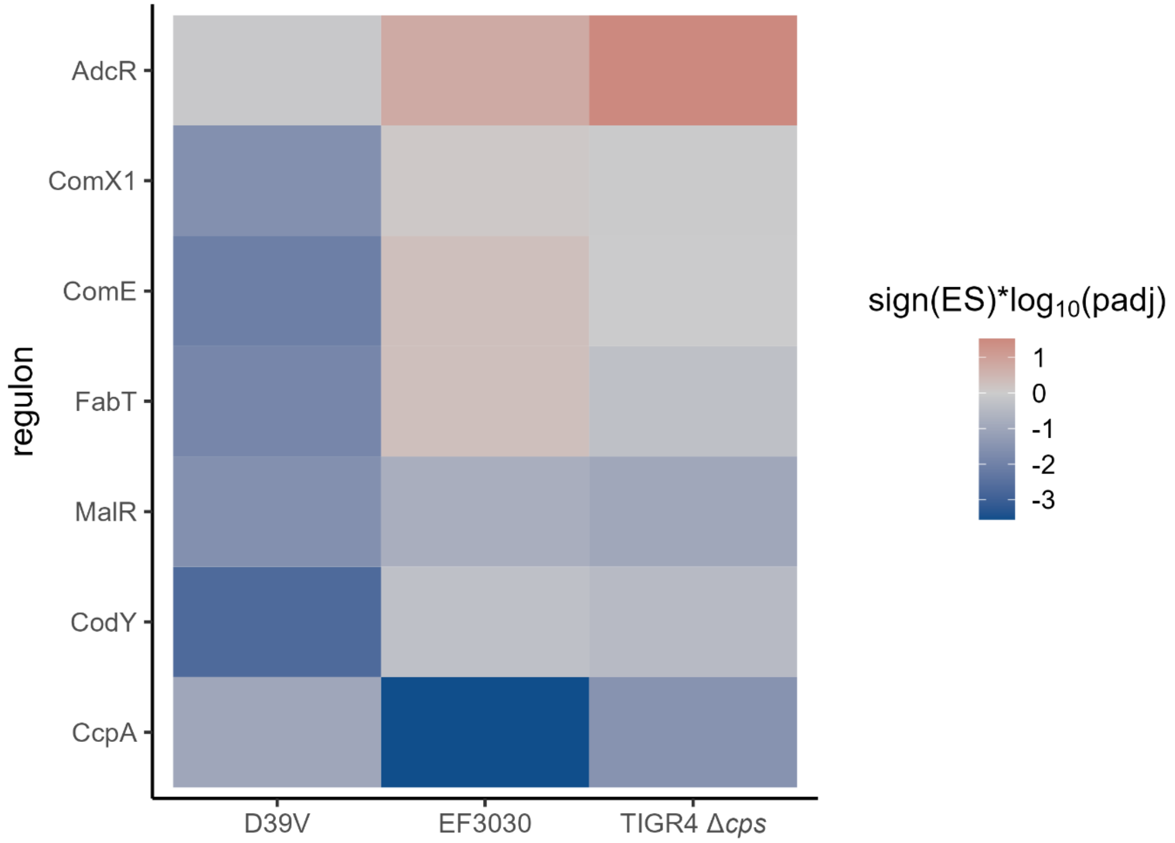

Supplementary Figure S6: Comparative analysis of the proteome profile changes from early exponential growth phase to later growth phases in CDM+. Proteome profiles of EF3030 and TIGR4Δ*cps* were compared at OD_{600} 1.0 to OD_{600} 0.4 and D39V was compared at OD_{600} 1.5 to OD_{600} 0.5. Proteome data of EF3030 was discussed in the presented study, proteome data of TIGR4Δ*cps* was obtained from an in-house pilot-study (unpublished data) and proteome data of D39V was obtained from the PRIDE Project PXD061622. For each strain growth dependent changes were tested using the ROPECA package. Gene set enrichment using the PneumoWiki regulon information was applied on the proteins. The resulting adjusted p-value (padj) was visualised and colouring was achieved according to the direction of the effect size (ES).

2 Supplementary Tables

Table S1: Pneumococcal and streptococcal strains used for studying the growth in the modified minimal medium (CDM+)

Species and strain	Relevant characteristics	Reference
S. pneumoniae		
D39	wild-type strain, serotype 2	NCTC 7466
D39*Δcps*	Allele exchange mutant *cps*::*aphA3* ,capsule deficient, Km^r	(Rennemeier et al. 2007)
TIGR4	wild-type strain, serotype 4	(Tettelin et al. 2001)
TIGR4*Δcps*	Allele exchange mutant *cps::aphA3* ,capsule deficient, Km^r	(Schulz et al. 2014)
EF3030	wild-type strain, serotype 19F	(Junges et al. 2019)
Invasive clinical isolate	8	RLS* (RWTH-Aachen)
Invasive clinical isolate	12F	RLS (RWTH-Aachen)
Invasive clinical isolate	22F	RLS (RWTH-Aachen)
Invasive clinical isolate	1	RLS (RWTH-Aachen)
Invasive clinical isolate	3	RLS (RWTH-Aachen)
Invasive clinical isolate	5	RLS (RWTH-Aachen)
Invasive clinical isolate	9N	RLS (RWTH-Aachen)
Invasive clinical isolate	7F	RLS (RWTH-Aachen)
Invasive clinical isolate	10A	RLS (RWTH-Aachen)
Invasive clinical isolate	11A	RLS (RWTH-Aachen)
Invasive clinical isolate	15B	RLS (RWTH-Aachen)
Invasive clinical isolate	17F	RLS (RWTH-Aachen)
Invasive clinical isolate	20	RLS (RWTH-Aachen)
Invasive clinical isolate	33F	RLS (RWTH-Aachen)
S. mutans	UA159	ATCC
S. suis	Strain 10, serotype 2	(Baums et al. 2009)
S. pyogenes	Group A Streptococcus 5448	(Kaul et al. 1997)
S. agalactiae	*S. agalactiae*, GBS STSS/NF-HH NSTI, STSS; Serotype Ib; ST8; Stockholm (Sweden)	(Sendi et al. 2009)

Km^r, kanamycin resistant; *RLS: Reference Laboratory for Streptococci

Table S2: Specific information of the Reversed phase liquid chromatography (RPLC)

instrument	Ultimate 3000 RSLC (Thermo Scientific)
trap column	75 µm inner diameter, packed with 3 µm C18 particles (Acclaim PepMap100, Thermo Scientific)
analytical column	Accucore 150-C18, (Thermo Fisher Scientific)<br>25 cm x 75 µm, 2,6 µm C18 particles, 150 Å pore size
buffer system	binary buffer system consisting of 0.1% acetic acid in HPLC-grade water (solvent A) and 100% ACN in 0.1% acetic acid (solvent B)
flow rate	300 nl/min
gradient	0min-2% B<br>2min-5% B<br>10min-7% B<br>70min-25% B<br>75min-40% B<br>77min-90% B<br>83min-90% B<br>85min-2% B<br>95min-2%B
column oven temperature	40°C

Table S3: Specific information of the Mass-spectrometry analysis in the data-independent acquisition mode

instrument	Orbitrap Exploris^{TM} 480
electrospray	Nanospray Flex^{TM} Ion Source
operation mode	data-independent
Full Scan Properties	
MS scan resolution	120000
AGC target	3e6 (300%)
maximum ion injection time for the MS scan	60 ms
Scan range	350 to 1200 m/z
Microscans	1
Polarity	positive
RF Lens	50%
Spectra data type	profile
DIA Properties (MS2)	
Resolution	30,000
maximum ion injection time for the MS/MS scans	auto
Normalized AGC target	3E6
Spectra data type	profile
Microscans	1
isolation window	66
Isolation window width	13 m/z
Window overlay	2 m/z
Fixed first mass	200
HCD collision energy	30%

Table S4: Proteins with significant changes in abundances during exponential growth.

The proteins identified in the cytosolic fraction and the supernatant fraction with a significant change in abundance during the late exponential growth (OD_{600nm} 1.0) compared to the early exponential growth (OD_{600nm} 0.4). The high abundant proteins and low abundant proteins detected in both the cytosolic and supernatant fractions are marked by “*”.

Table S4A: High abundant proteins

		Locus tag	protein symbol	protein description	fold change
**Cytosol**	transporters	EF3030_RS09905	BguD	PTS transporter subunit EIIC	2.4
		EF3030_RS09915	BguC	PTS lactose/cellobiose transporter subunit IIA	2.0
		EF3030_RS09910	BguB	PTS sugar transporter subunit IIB	1.8
		EF3030_RS06705	ABC-N/P_3	ABC transporter ATP-binding protein	3.9
		EF3030_RS03710	ABC-NBD_6	ABC transporter ATP-binding protein	1.6
		* EF3030_RS08090	ABC-NBD_19	ABC transporter ATP-binding protein	2.0
		EF3030_RS07180	AliB	peptide ABC transporter substrate-binding protein	1.5
		* EF3030_RS10730	AdcC	metal ABC transporter ATP-binding protein	3.5
		* EF3030_RS10720	AdcA	zinc ABC transporter substrate-binding lipoprotein AdcA	2.9
		* EF3030_RS04830	AdcAII	zinc-binding lipoprotein AdcAII	10.6
		* EF3030_RS03430	CtpA	heavy metal translocating P-type ATPase	4.2
		EF3030_RS03715	YknZ	ABC transporter permease	1.7
	gene regulators	EF3030_RS03420	CopY	CopY/TcrY family copper transport repressor	5.8
		EF3030_RS08085	GntR	GntR family transcriptional regulator	1.9
		* EF3030_RS10735	AdcR	zinc-dependent transcriptional regulator AdcR	5.6
	others	EF3030_RS07410	Dpr	Dps family protein	2.1
		EF3030_RS06080	DprA	DNA-processing protein DprA	4.5
	metabolism	* EF3030_RS06920	Nfr1	NAD(P)H-dependent oxidoreductase	1.7
		* EF3030_RS06915	Nfr2	NADPH-dependent FMN reductase	1.5
		EF3030_RS10225	GuaA_2	gamma-glutamyl-gamma-aminobutyrate hydrolase family protein	2.5
		EF3030_RS08670	EF3030_RS08670	PEP phosphonomutase	2.2
		* EF3030_RS09900	BguA	glycoside hydrolase family 1 protein	1.8
		* EF3030_RS02000	Mip	carboxymuconolactone decarboxylase family protein	2.3
		* EF3030_RS06675	YdcP_2	U32 family peptidase	1.7

	cell structure/	*EF3030_RS05355	SrtA	sortase SrtA	1.5
	competence	EF3030_RS09295	SsbB	single-stranded DNA-binding protein	6.8
		* EF3030_RS10050	ComGA	competence type IV pilus ATPase ComGA	4.7
	virulence	* EF3030_RS05555	PhtD/PhtB	pneumococcal-type histidine triad protein	33.4
		* EF3030_RS04835	PhtD	pneumococcal histidine triad protein PhtD	12.3
		* EF3030_RS04840	PhtE	pneumococcal histidine triad protein PhtE	11.2
		* EF3030_RS06220	LytA_3	caspase family protein	5.0
		* EF3030_RS06215	LytB_4	N-acetylmuramoyl-L-alanine amidase family protein	3.5
	unknown	EF3030_RS06435	EF3030_RS06435	hypothetical protein	1.5
		EF3030_RS09985	EF3030_RS09985	hypothetical protein	1.5
**Supernatant**	transporters	* EF3030_RS10730	AdcC	metal ABC transporter ATP-binding protein	2.5
		* EF3030_RS10720	AdcA	zinc ABC transporter substrate-binding lipoprotein AdcA	2.3
		* EF3030_RS04830	AdcAII	zinc-binding lipoprotein AdcAII	6.4
		* EF3030_RS08090	ABC-NBD_19	ABC transporter ATP-binding protein	1.7
		* EF3030_RS03430	CtpA	heavy metal translocating P-type ATPase	3.4
	gene regulator s	* EF3030_RS10735	AdcR	zinc-dependent transcriptional regulator AdcR	2.9
	others	EF3030_RS03100	MsrAB_1	redoxin family protein	1.8
		EF3030_RS08795	PfbA	multi-ligand-binding adhesin PfbA	1.8
	metabolism	* EF3030_RS06920	Nfr1	NAD(P)H-dependent oxidoreductase	1.6
		* EF3030_RS06915	Nfr2	NADPH-dependent FMN reductase	1.8
		EF3030_RS04780	MtnN	5'-methylthioadenosine/adenosylhomocysteine nucleosidase	1.9
		* EF3030_RS09900	BguA	glycoside hydrolase family 1 protein	2.0
		EF3030_RS00045	EF3030_RS00045	serine hydrolase	1.9
		* EF3030_RS02000	Mip	carboxymuconolactone decarboxylase family protein	1.8
	cell structure/ surface	EF3030_RS01780	Eng	SpGH101 family endo-alpha-N-acetylgalactosaminidase	1.7
	co m	EF3030_RS00240	ComB	competence pheromone export protein ComB	6.1

	* EF3030_RS10050	ComGA	competence type IV pilus ATPase ComGA	5.5
virulence	EF3030_RS10895	CbpD	choline binding-anchored murein hydrolase CbpD	54.7
	EF3030_RS10555	PcpA	choline-binding protein PcpA	1.6
	EF3030_RS00555	EF3030_RS00555	lactococcin 972 family bacteriocin	1.6
	EF3030_RS02515	BlpB	bacteriocin secretion accessory protein	1.7
	EF3030_RS02525	BlpU	bacteriocin-like peptide BlpU	4.5
	EF3030_RS05665	ZmpA	ZmpA/ZmpB/ZmpC family metallo-endopeptidase	1.6
	* EF3030_RS05555	PhtD/PhtB	pneumococcal-type histidine triad protein	6.9
	* EF3030_RS04835	PhtD	pneumococcal histidine triad protein PhtD	5.3
	* EF3030_RS04840	PhtE	pneumococcal histidine triad protein PhtE	13.9
	* EF3030_RS06220	LytA_3	caspase family protein	6.1
	* EF3030_RS06215	LytB_4	N-acetylmuramoyl-L-alanine amidase family protein	3.9
	EF3030_RS03125	ZmpB	ZmpA/ZmpB/ZmpC family metallo-endopeptidase	1.8
	EF3030_RS05660	ZmpD	SIALI-17 repeat-containing surface protein	2.0
unknown	EF3030_RS10670	EabC	glycoside hydrolase family 98 domain-containing protein	1.7
	EF3030_RS04325	EF3030_RS04325	TIGR03943 family protein	1.7
	EF3030_RS04305	EF3030_RS04305	DUF4300 family protein	1.9
	EF3030_RS02360	EndoD	bacterial Ig-like domain-containing protein	2.2

Table S4B: Low abundant proteins

		Locus tag	protein symbol	protein description	fold change
cytosol	transporters	EF3030_RS03565	MalT	PTS transporter subunit IIBC	-1.5
		EF3030_RS03030	PTS-EII_11	PTS sugar transporter subunit IIA	-1.6
		EF3030_RS10875	ThiZ	ABC transporter ATP-binding protein	-1.6
		EF3030_RS00880	EF3030_RS00880	NRAMP family divalent metal transporter	-1.7
		EF3030_RS03040	PTS-EII_12	PTS galactitol transporter subunit IIC	-1.8
		EF3030_RS07930	SatA	ABC transporter substrate-binding protein	-1.9
		EF3030_RS07920	SatC	carbohydrate ABC transporter permease	-1.9
		EF3030_RS07935	NanP	PTS transporter subunit EIIC	-1.9
		EF3030_RS03035	EF3030_RS03035	PTS sugar transporter subunit IIB	-2.1
		EF3030_RS05495	LacF-2	PTS lactose/cellobiose transporter subunit IIA	-2.5
		EF3030_RS05500	LacE-2	lactose-specific PTS transporter subunit EIIC	-2.6
		EF3030_RS00470	ABC-SBP_1	ABC transporter substrate-binding protein	-2.7
		EF3030_RS07965	ABC-SBP_7	sugar ABC transporter substrate-binding protein	-2.9
		* EF3030_RS04170	FruA	fructose-specific PTS transporter subunit EIIC	-1.9
		* EF3030_RS09050	TreP	PTS system trehalose-specific EIIBC component	-2.8
		* EF3030_RS00340	GadW	PTS mannose/fructose/sorbose/N-acetylgalactosamine transporter subunit IIC	-3.1
		EF3030_RS10400	MalX	maltodextrin ABC transporter substrate-binding protein	-1.6
	gene regulators	EF3030_RS08905	NmlR	stress response transcriptional regulator NmlR	-1.6
		EF3030_RS10810	EF3030_RS10810	helix-turn-helix domain-containing protein	-1.6
		EF3030_RS04160	FruR	DeoR/GlpR family DNA-binding transcription regulator	-2.6
		EF3030_RS05490	LacT	transcription antiterminator	-3.4
	metabolism	EF3030_RS10605	ArcA	arginine deiminase	-2.5
		EF3030_RS10615	ArcC	carbamate kinase	-2.2
		EF3030_RS10610	ArgF/ArcB	ornithine carbamoyltransferase	-1.8
		EF3030_RS10395	MalQ	4-alpha-glucanotransferase	-1.5
		EF3030_RS00330	BgaC	beta-galactosidase	-1.5
		EF3030_RS10390	GlgP	glycogen/starch/alpha-glucan family phosphorylase	-1.7
		EF3030_RS10575	GcnA	beta-N-acetylhexosaminidase	-2.8
		EF3030_RS01435	AdhP	alcohol dehydrogenase AdhP	-1.6

	EF3030_RS10060	Adh	zinc-dependent alcohol dehydrogenase family protein	-1.6
	EF3030_RS03050	BgaA	LPXTG-anchored adhesin/beta-galactosidase BgaA	-1.8
	EF3030_RS00540	SdaAB	L-serine ammonia-lyase, iron-sulfur-dependent subunit beta	-1.8
	EF3030_RS10280	PstS1	substrate-binding domain-containing protein	-1.9
	EF3030_RS08885	GalT-2	UDP-glucose--hexose-1-phosphate uridylyltransferase	-2.1
	EF3030_RS07940	NanE-1	N-acetylmannosamine-6-phosphate 2-epimerase	-2.1
	EF3030_RS08890	GalK	galactokinase	-2.2
	EF3030_RS05510	LacG	6-phospho-beta-galactosidase	-2.3
	EF3030_RS04165	PfkB	1-phosphofructokinase	-2.3
	EF3030_RS10805	GlpK	glycerol kinase GlpK	-2.6
	EF3030_RS10800	GlpO	type 1 glycerol-3-phosphate oxidase	-2.8
	EF3030_RS07945	IolX_2	Gfo/Idh/MocA family oxidoreductase	-2.8
	EF3030_RS09040	TreC	alpha,alpha-phosphotrehalase	-2.0
	EF3030_RS05540	NrdH	glutaredoxin-like protein NrdH	-2.0
	EF3030_RS06445	Amy	alpha-amylase	-2.1
	EF3030_RS10585	MngB	alpha-mannosidase	-2.6
cell surface/ surface modification	EF3030_RS07485	MurT	MurT ligase domain-containing protein	-1.5
	EF3030_RS07895	NanA_2	dihydrodipicolinate synthase family protein	-1.8
	EF3030_RS10975	PcsB	CHAP domain-containing protein	-1.5
	EF3030_RS00320	StrH	LPXTG-anchored beta-N-acetylhexosaminidase StrH	-2.3
virulence	EF3030_RS07975	NanA	SIALI-17 repeat-containing surface protein	-2.8
	EF3030_RS01645	LuxS	S-ribosylhomocysteine lyase	-1.5
unknown	EF3030_RS00480	EF3030_RS00480	DUF4299 family protein	-1.6
	EF3030_RS07915	YjgK	YhcH/YjgK/YiaL family protein	-1.7
	EF3030_RS00355	AgaS	SIS domain-containing protein	-1.8
	EF3030_RS08780	EF3030_RS08780	DUF1868 domain-containing protein	-2.0
	EF3030_RS07890	NanK	ROK family protein	-2.0
others	EF3030_RS03025	PrtA	S8 family serine peptidase	-1.6
	EF3030_RS05310	MutY	A/G-specific adenine glycosylase	-1.6
	EF3030_RS10075	Tgt	tRNA guanosine(34) transglycosylase Tgt	-1.7
	EF3030_RS10625	PepV	dipeptidase	-1.8
	EF3030_RS01000	EF3030_RS01000	SP_0198 family lipoprotein	-1.8

		EF3030_RS01790	RecU	Holliday junction resolvase RecU	-2.4
		EF3030_RS10100	EF3030_RS10100	LysM domain-containing protein	-3.1
		EF3030_RS07825	YlmH	RNA-binding protein	-1.6
**supernatant**	transporters	EF3030_RS10405	MalC	sugar ABC transporter permease	-1.6
		EF3030_RS02950	BrnQ	branched-chain amino acid transport system II carrier protein	-1.6
		* EF3030_RS04170	FruA	fructose-specific PTS transporter subunit EIIC	-1.6
		* EF3030_RS09050	TreP	PTS system trehalose-specific EIIBC component	-1.5
		* EF3030_RS00340	GadW	PTS mannose/fructose/sorbose/N-acetylgalactosamine transporter subunit IIC	-1.7
	unknown	EF3030_RS07970	TabA_2	YhcH/YjgK/YiaL family protein	-1.7
	others	EF3030_RS08270	YqeH	ribosome biogenesis GTPase YqeH	-1.5
		EF3030_RS09635	RsgA	ribosome small subunit-dependent GTPase A	-1.6
		EF3030_RS10370	RrmA	methyltransferase domain-containing protein	-1.6
		EF3030_RS11110	ParB	ParB/RepB/Spo0J family partition protein	-1.6
		EF3030_RS07470	CshA	DEAD/DEAH box helicase	-1.7

References

Baums, Christoph Georg; Kock, Christoph; Beineke, Andreas; Bennecke, Katharina; Goethe, Ralph; Schröder, Charlotte et al. (2009): *Streptococcus suis* bacterin and subunit vaccine immunogenicities and protective efficacies against serotypes 2 and 9. In: *Clinical and vaccine immunology : CVI* 16 (2), S. 200–208. DOI: 10.1128/CVI.00371-08.

Junges, R.; Maienschein-Cline, M.; Morrison, D. A.; Petersen, F. C. (2019): Complete Genome Sequence of *Streptococcus pneumoniae* Serotype 19F Strain EF3030. In: *Microbiology resource announcements* 8 (19). DOI: 10.1128/MRA.00198-19.

Kaul, R.; McGeer, A.; de Low; Green, K.; Schwartz, B.; Simor, A. E. (1997): Population-Based Surveillance for Group A Streptococcal Necrotizing Fasciitis: Clinical Features, Prognostic Indicators, and Microbiologic Analysis of Seventy-Seven CasesStreptococcus pneumoniae: Genetic requirements.

Rennemeier, Claudia; Hammerschmidt, Sven; Niemann, Silke; Inamura, Seiichi; Zähringer, Ulrich; Kehrel, Beate E. (2007): Thrombospondin-1 promotes cellular adherence of gram-positive pathogens via recognition of peptidoglycan. In: *FASEB journal : official publication of the Federation of American Societies for Experimental Biology* 21 (12), S. 3118–3132. DOI: 10.1096/fj.06-7992com.

Schulz, Christian; Gierok, Philipp; Petruschka, Lothar; Lalk, Michael; Mäder, Ulrike; Hammerschmidt, Sven (2014): Regulation of the arginine deiminase system by ArgR2 interferes with arginine metabolism and fitness of Streptococcus pneumoniae. In: *mBio* 5 (6). DOI: 10.1128/mBio.01858-14.

Sendi, Parham; Johansson, Linda; Dahesh, Samira; Van-Sorge, Nina M.; Darenberg, Jessica; Norgren, Mari et al. (2009): Bacterial phenotype variants in group B streptococcal toxic shock syndrome. In: *Emerging infectious diseases* 15 (2), S. 223–232. DOI: 10.3201/eid1502.080990.

Tettelin, H.; Nelson, K. E.; Paulsen, I. T.; Eisen, J. A.; Read, T. D.; Peterson, S. et al. (2001): Complete genome sequence of a virulent isolate of Streptococcus pneumoniae. Science.
